# Fresh Dairy Quality in the Era of Smart Food Systems: Deterioration Pathways, Analytical Technologies, and Predictive Modeling

**DOI:** 10.3390/foods15193439

**Published:** 2026-09-26

**Authors:** Laíres Lima, Ana I. Pereira, Manuela Pintado, Márcio Carocho

**Affiliations:** 1 CIMO, LA SusTEC, Campus de Santa Apolónia, Bragança Polytechnic University, 5300-253 Bragança, Portugal; laires.lima@ipb.pt; 2 Research Centre in Digitalization and Intelligent Robotics (CeDRI), Associate Laboratory for Sustainability and Technology in Mountains Regions (SusTEC), Campus de Santa Apolónia, Bragança Polytechnic University, 5300-253 Bragança, Portugal; apereira@ipb.pt; 3 CBQF—Centro de Biotecnologia e Química Fina—Laboratório Associado, Escola Superior de Biotecnologia, Universidade Católica Portuguesa, Rua Diogo Botelho 1327, 4169-005 Porto, Portugal; mpintado@ucp.pt

**Keywords:** fresh dairy products, shelf-life prediction, quality endpoints, quality monitoring, predictive modeling, machine learning, analytical technologies

## Abstract

This review critically examines product-specific deterioration pathways in fresh dairy products and evaluates how analytical technologies, kinetic models, chemometric tools, and machine learning approaches support quality assessment and shelf-life prediction. Current evidence shows that many studies identify storage-related changes; however, few can predict the point at which a product reaches its acceptability limit and end of shelf-life. Reliable shelf-life prediction therefore requires a clear definition of the dominant deterioration pathway, a representative quality indicator, and a well-defined microbiological, physicochemical, structural, or sensory acceptability threshold. The analytical signal must demonstrate a validated mechanistic or predictive relationship with the selected quality threshold, rather than merely correlate with storage duration. Adopting an endpoint-centered framework would enable a more efficient integration of deterioration mechanisms, analytical measurements, and predictive modeling approaches, thereby providing a stronger scientific basis for shelf-life determination and quality management in fresh dairy products. Future progress will depend on the generation of representative multi-batch datasets, independent validation, realistic time–temperature histories, and model evaluation across diverse storage and distribution scenarios.

## 1. Introduction

Fresh refrigerated dairy products have considerable nutritional, commercial, and sensory value. Their high water activity, nutrient-rich composition, and relatively mild processing conditions also contribute to microbial activity and several forms of biochemical and physicochemical deterioration [1,2,3]. The relative importance of these changes varies with the product matrix, initial microbiota, processing history, packaging conditions, and temperature exposure.

Many models have been proposed for shelf-life prediction, but their reliability in practice remains limited. A common problem is that they predict changes during storage without defining when the product becomes unacceptable. Reliable prediction requires a measurable indicator that is linked to a clear quality limit and remains valid across different batches and storage conditions.

Advanced analytical technologies can detect chemical and structural changes that conventional measurements may not capture with the same speed or level of detail. Spectroscopic, imaging, and sensor-based methods provide analytical fingerprints of dairy product quality [4,5,6]. Chemometric and machine learning methods can relate these signals to quality attributes and microbial behavior [7,8,9]. Instrumental measurements can also reveal processing-related structural changes during fresh-cheese storage [10]. Their practical value, however, depends on the relevance of the predicted outcome, the representativeness of the data, and evidence of performance beyond the original dataset [11].

Reviews have examined machine learning applications across the dairy sector [7], artificial-intelligence approaches to shelf-life prediction across food categories [11], and the distinction between storage stability and shelf-life prediction in microbially stabilized dairy products [12]. The present review focuses specifically on fresh refrigerated dairy products and applies an endpoint-centered framework linking product-specific deterioration pathways and analytical signals to explicit quality or safety criteria, time-to-failure estimation, and independent validation. This framework distinguishes storage monitoring, quality classification, and intermediate-response prediction from direct shelf-life prediction and evaluates the extent to which these approaches have been validated under realistic production and cold-chain conditions.

## 2. Review Approach

### 2.1. Review Design and Scope

This study was conducted as a critical narrative review supported by a structured literature search. The structured search and documented study-selection process were used to provide a transparent basis for the construction of the evidence base while retaining the interpretative and integrative scope of a narrative review. Record identification, deduplication, screening, full-text assessment, and study inclusion were therefore documented systematically, without classifying the review as a systematic review.

The review focused on fresh refrigerated dairy products and examined deterioration processes, analytical technologies, kinetic and probabilistic models, chemometric methods, and machine learning approaches according to their contribution to quality, safety, and shelf-life assessment.

Fresh dairy products were defined as refrigerated milk and dairy products consumed without prolonged ripening or maturation and whose quality and shelf-life are primarily dependent on refrigerated storage. Eligible matrices included milk, yogurt, kefir, ricotta, mozzarella, paneer, chhana, quark, cottage cheese, fresh cheese, fermented milk, and lassi. Ripened or aged cheeses, dried dairy products, milk and whey powders, UHT or sterilized products, frozen dairy products, and other shelf-stable products were excluded.

### 2.2. Literature Search Strategy

Scopus and Web of Science Core Collection were searched for studies published from 2015 to 2025. The final search was performed on 8 August 2026. Eligibility was restricted to English-language articles, but no document-type filter was applied during the database searches to avoid prematurely excluding potentially relevant records. The search combined terms related to dairy matrices, shelf-life and storage-related deterioration, and analytical or modeling approaches. Searches were conducted in the TITLE-ABS-KEY fields in Scopus and the Topic (TS) field in Web of Science. The complete database-specific Boolean search strategies, search fields, and restrictions are provided in Supplementary Table S1. The searches retrieved 6898 records: 3599 from Scopus and 3299 from Web of Science. Biblioshiny, the graphical interface of the bibliometrix R package (version 5.5.0), run within RStudio (version 2026.06.0+242), was used to import and merge the records, remove duplicates, and support title and abstract screening.

### 2.3. Study Selection

During screening, original experimental studies were considered eligible for the primary narrative synthesis when they were conducted in a real dairy matrix and investigated a measured or model-derived response related to quality, safety, deterioration, freshness, stability, or shelf-life assessment. Reviews, editorials, commentaries, conference abstracts, and purely theoretical publications were excluded from the primary study set. Relevant reviews and methodological papers were retained separately as background or supporting references and were not counted among the included primary studies.

Studies were also excluded when the dairy product or analytical/modeling approach was outside the predefined scope, no direct relationship with quality, safety, deterioration, or shelf-life was established, storage conditions or experimental design were insufficiently described, or the study was not conducted in a real dairy matrix.

Before screening, 2129 duplicate records were removed. A rule-based R script was then applied to the bibliographic records imported with bibliometrix to flag potentially ineligible records by searching titles, abstracts, and keywords for terms corresponding to the excluded product categories defined above. Flagged records were manually checked against these criteria, resulting in the removal of 192 records. The remaining 4577 records were screened by title and abstract, and 4186 were excluded.

The 391 remaining reports were assessed in full text. Of these, 345 were excluded: 83 because the dairy product was outside the predefined scope, 10 because no direct relationship with quality, safety, deterioration, or shelf-life was established, 194 because the analytical or modeling approach was outside the review scope, 7 because storage conditions or experimental design were insufficiently described, 16 because the study was not conducted in a real dairy matrix, and 35 because the publication or study type was ineligible. The final evidence base comprised 46 primary studies. The study-selection process is summarized in Figure 1.

### 2.4. Evidence Mapping

The 46 included studies were mapped according to the dairy matrix, analytical or modeling approach, measured or predicted response, endpoint or criterion, relationship with shelf-life, and validation strategy. This classification was used to distinguish studies that primarily monitored storage-related changes from those that predicted intermediate quality outcomes or directly estimated a defined shelf-life endpoint. The study-level evidence map, including reported predictive performance where applicable, is provided in Supplementary Table S2.

Nine studies (19.6%) primarily monitored storage-related changes or identified potential deterioration markers, while 18 (39.1%) predicted storage age, intermediate quality responses, quality indices, or current quality classes. Thirteen studies (28.3%) determined or predicted shelf-life using an explicit quality-related endpoint; nine of these modeled the time required to reach the endpoint, whereas four determined shelf-life empirically from predefined failure criteria. Two additional studies (4.3%) estimated the probability of a defined spoilage event without directly predicting time to failure, and four (8.7%) primarily addressed safety-related outcomes.

Independent validation was considered separately from the prediction target. Thirty studies (65.2%) did not include independent evaluation beyond model development, six (13.0%) used internal cross-validation or a hold-out set from the same experimental dataset, seven (15.2%) used independent samples or production batches, and three (6.5%) were evaluated using external commercial or industrial data or an independently generated dynamic-storage scenario. Thus, 10 of the 46 studies (21.7%) included validation beyond the original model-development dataset.

The quality and reporting of the review were self-assessed using the Scale for the Assessment of Narrative Review Articles (SANRA) [13], which was developed specifically for narrative reviews. The completed SANRA self-assessment is provided in Supplementary Table S3.

## 3. Quality Loss in Fresh Dairy Products

Quality loss in fresh dairy products results from the combined effects of microbial, enzymatic, chemical, physical, and sensory changes, with no single deterioration reaction acting as the sole cause. These processes may occur simultaneously but progress at different rates depending on product composition, initial microbiota, processing history, packaging, oxygen or light exposure, storage temperature, and storage time (Figure 2) [1,2].

For shelf-life assessment, the relevant deterioration pathway is the one that first causes the product to exceed a defined acceptability limit under its intended storage and use conditions. This pathway may not produce the largest analytical change.

Microbial growth is a major cause of deterioration in refrigerated pasteurized milk. Psychrotrophic bacteria can grow at refrigeration temperatures, and post-pasteurization contamination may introduce additional spoilage microorganisms whose growth depends strongly on temperature [14,15]. The effects extend beyond viable cell counts. Psychrotrophic bacteria can produce extracellular proteases, lipases, and phospholipases, some of which remain active after heat treatment has inactivated the bacterial cells [15]. Proteolysis and lipolysis may thus continue during storage and contribute to quality loss even when viable populations are low at the time of analysis. Raw-milk studies have also shown regional and seasonal variation in lipase activity and identified ionic calcium as a potential indicator of milk-fat stability [16]. In pasteurized buffalo milk, plant-leaf extracts delayed microbial, acidity, and enzyme-related failure criteria, extending the reported shelf-life from 5 to 16 days at the highest concentration [17].

Microbial counts do not always reflect the extent of sensory deterioration. Associations have been reported among total microbial populations, volatile compounds, and sensory attributes in pasteurized milk, although cell counts explained only part of the observed variation. This incomplete correspondence was linked to differences in the spoilage potential and metabolic activity of the contaminating microorganisms [18]. Another study found relationships among bacterial growth, protease and lipase activities, and volatile formation at different storage temperatures [19]. *Pseudomonas* spp. may also promote lipolysis and the accumulation of free fatty acids associated with undesirable flavors [20]. Microbial enumeration is thus informative but may not fully describe the sensory consequences of deterioration.

Fermented dairy products have a different combination of failure mechanisms. In yogurt, starter cultures may remain active during refrigerated storage, leading to continued acidification. Higher acidity can coincide with lower viscosity and sensory scores, and all three responses are influenced by storage temperature [3]. However, physicochemical changes vary among yogurt matrices. Cow- and goat-milk yogurts may show different pH patterns, while syneresis can remain relatively stable during storage [21]. Acidity, texture, and whey separation consequently provide complementary, non-interchangeable information about deterioration.

Fungal spoilage may limit yogurt shelf-life even when post-acidification remains acceptable. In experimentally contaminated Greek yogurt, yeasts and molds reached spoilage-associated populations and caused visible mold growth, gas formation, and surface changes [22]. Post-pasteurization processing environments are also potential sources of fungal contamination. The extent of yeast growth during storage depends on the species, temperature, initial contamination level, and presence of bioprotective cultures [23].

Several processes may also compete to limit the shelf-life of fresh cheeses. Ricotta is particularly susceptible to microbial deterioration because of its high water activity and relatively high pH [24]. Packaging affects the rates of these changes through differences in oxygen and water-vapor permeability, which influence microbial growth, volatile formation, and sensory acceptability. Under some conditions, the microbiological limit is reached before sensory rejection occurs [24]. Studies based on both microbiological and sensory criteria have likewise shown that estimated ricotta shelf-life depends on which limit is reached first [25]. Product-specific failure criteria are therefore necessary because the attribute that limits shelf-life can differ among fresh cheeses. Industrial ricotta packaged under modified atmosphere nevertheless developed high Pseudomonas and Enterobacteriaceae populations during 21 days of storage, showing that packaging alone did not compensate for post-process contamination [26]. In quark cheese, dense-phase carbon dioxide treatment kept treated samples within the reported shelf-life through day 14, whereas the untreated control became unsuitable because of excessive yeast [27]. Active salicylate packaging extended modeled mozzarella shelf-life by approximately 2 days at 4 °C [28].

Storage conditions can alter both the rate and the main pathway of deterioration. Light exposure is especially important for milk sold in transparent packaging. Pasteurized milk in clear PET remains relatively stable in the dark, whereas retail-display lighting accelerates riboflavin degradation, changes the volatile profile, and causes progressive sensory deterioration [29]. In ricotta, packaging permeability modifies the internal oxygen and water-vapor conditions, affecting microbial and chemical changes during storage [24]. Shelf-life estimates obtained under one packaging or retail condition may not remain valid when those conditions change.

Temperature influences many of these processes. In pasteurized milk, higher storage temperatures accelerate microbial growth and shorten the time needed to reach spoilage-associated populations [15]. Metabolomic analyses have also identified compositional changes linked to storage time and temperature [30]. Temperature history becomes especially important when refrigeration fluctuates. Under inadequate refrigeration, mozzarella develops marked changes in succinate, acetate, and lactate, while conventional measurements such as pH, fat, and protein provide less separation between storage conditions. After four weeks, succinate concentrations under inadequate refrigeration were similar to those measured at the stated expiry date under adequate refrigeration, indicating faster chemical deterioration [31]. Constant-temperature experiments remain useful for kinetic characterization but cannot reproduce every change encountered during distribution, retail, and domestic storage.

No single measurement can capture deterioration across all fresh dairy products. Microbial counts, acidity, texture, volatile compounds, metabolites, instrumental responses, and sensory scores represent different aspects of quality, and their trajectories may diverge during storage. Their value in shelf-life assessment depends on how closely they reflect the process that limits the product under the conditions studied. A response may track storage time reliably and still serve only as a quality-monitoring indicator, without defining when the product becomes unacceptable.

Quality deterioration must also be distinguished from microbiological safety. Models of Listeria monocytogenes growth or survival in fresh soft cheese and paneer describe safety-related outcomes, not sensory or commercial deterioration [32,33]. Conversely, microbial spoilage, proteolysis, lipolysis, volatile formation, and sensory rejection do not demonstrate the presence of a food-safety hazard. A product may consequently require separate quality and safety criteria, even when both respond to the same storage conditions.

Shelf-life prediction should begin by identifying the deterioration process most likely to limit the product under its intended storage conditions. A representative response and the value at which it indicates product failure must then be defined. This distinction between a storage-related change and a failure criterion provides the basis for endpoint selection and model development. Figure 3 summarizes this framework, from failure definition to predictive modeling and external validation.

## 4. Kinetic Principles of Quality Deterioration and Shelf-Life

Shelf-life models have two main functions. They can describe changes in microbial, chemical, physicochemical, structural, or sensory responses during storage, or estimate when those responses reach a level associated with product failure. These functions are related but not equivalent. A kinetic equation may reproduce a storage trajectory accurately without indicating when the product becomes unacceptable. A failure criterion and a measurable response linked to that criterion must therefore be defined before selecting the mathematical model.

### 4.1. Selection of Quality Indicators and Endpoints

In this review, an endpoint is a predefined criterion that indicates when a product becomes unacceptable in terms of quality or no longer meets a microbiological safety requirement. Quality endpoints may include sensory rejection or specified limits for spoilage-related microbial populations, physicochemical properties, or structural changes. Safety endpoints are based on explicit microbiological criteria. Models of pathogen growth, survival, or persistence describe safety-related processes but establish an endpoint only when their predictions are evaluated against a defined limit. Keeping these concepts separate prevents storage-related changes or pathogen behavior from being misinterpreted as direct measures of sensory or commercial shelf-life.

Microbial thresholds illustrate this distinction. Pasteurized milk has been considered expired when total plate counts exceeded 4.47 log CFU/mL, with shelf-life calculated as the time required to reach this concentration [34]. A limit of 20,000 CFU/mL has also been used as a conservative quality criterion for spoilage associated with post-pasteurization contamination [14]. In yogurt simulations, products were classified as spoiled when yeast populations exceeded 5 log CFU/mL [23]. However, this threshold was assumed to correspond to visible spoilage; the population at which spoilage became detectable was not experimentally established for the four yeast strains evaluated. Including a microbial limit in a model does not, on its own, make that value a validated rejection threshold.

Sensory criteria define failure differently. In ricotta, an overall quality score of 4 on a seven-point scale was set as the minimum acceptable value. Microbiological and sensory data were modeled separately to estimate the corresponding acceptability limits [25], and shelf-life was defined by the limit reached first. Another ricotta study evaluated microbiological criteria together with an overall acceptability threshold of 2.8. Under the packaging conditions tested, microbial growth became limiting before sensory rejection [24]. In yogurt, acidity, viscosity, and sensory criteria have also been modeled separately, with the earliest predicted failure time defining product acceptability [3]. When several endpoints are used, shelf-life is determined by the first predefined limit reached, irrespective of which attribute changes most markedly.

Quality indicators serve a different purpose. They monitor deterioration but define product failure only when an appropriate limit has been established. This distinction is particularly relevant in analytical and chemometric studies. UV–Vis spectra have been used to predict yogurt storage age with an external test set, showing that the spectral response changed systematically during storage [35]. However, the model predicted storage duration, not a defined acceptability endpoint. Metabolomic profiles have also distinguished ricotta samples by storage time, using the shelf-life time point as the class variable [36]. These results document storage-related chemical changes but do not determine when product failure occurs.

Derived quality indices require the same caution. A biogenic-amine-based index has been associated with physicochemical and sensory changes in yogurt during storage [21]. Direct use of this index for shelf-life prediction would require a defined value corresponding to unacceptable quality. Storage-age prediction, quality classification, candidate-marker identification, and endpoint-based shelf-life prediction are distinct modeling objectives and should be interpreted accordingly. A quality-index model for date-syrup-fortified laban followed physicochemical and sensory changes during seven days of storage and was predicted from NIR spectra, but it did not define an independent rejection threshold [37].

The interpretation of microbiological models also depends on the microorganism and criterion represented. Chhana shelf-life has been estimated from several microbial groups, with total plate counts reaching the defined critical concentration before coliforms or yeasts and molds under the conditions studied [38]. These are quality-related spoilage outcomes. By comparison, models of *Listeria monocytogenes* growth and growth/no-growth boundaries in paneer, along with survival models for *L. monocytogenes* and *Escherichia coli* O157:H7 in refrigerated kefir, address microbiological safety [33,39]. Spoilage and safety responses may be evaluated under the same temperature history, as in studies of *L. monocytogenes and Pseudomonas putida* in pasteurized milk, but each requires a separate interpretation [40].

The failure event should guide model selection. Continuous quality indicators can be described with kinetic equations, microbial populations with growth or survival functions, and variation in contamination, temperature, or growth with stochastic approaches. These models estimate changes in a response; they do not define shelf-life unless that response is evaluated against a specified criterion. When several valid quality criteria are available, the first limit reached determines product life. Safety must be assessed separately because acceptable sensory or commercial quality does not guarantee microbiological safety.

### 4.2. Kinetic and Probabilistic Models for Endpoint-Based Shelf-Life Prediction

Kinetic and probabilistic models describe different aspects of deterioration. Reaction-order models represent changes in continuous quality responses; temperature-dependence models describe how rates vary with storage conditions; primary and secondary microbial models represent population dynamics and their environmental dependence; survival models characterize persistence or inactivation; and stochastic models incorporate variability in the factors controlling spoilage or safety. Their contribution to shelf-life assessment depends on how the modeled response is connected to a defined endpoint. These models are show in Table 1.

Reaction-order models are suitable when a quality response changes systematically during storage. In yogurt, acidity, viscosity, and sensory deterioration have been modeled separately and converted into shelf-life estimates using predefined limits. At 5 °C, each response produced a different failure time, and the shortest was used as the final shelf-life estimate [3]. The kinetic models were thus directly connected to explicit failure criteria.

Other applications require more cautious interpretation. First-order betalain degradation and zero-order changes in yogurt color have been modeled together with activation energy and Q_10_ values to describe temperature dependence [38]. These models quantify pigment and color stability but cannot define the end of yogurt shelf-life without a product-level rejection criterion. Reaction-order models have also been fitted to sensory, physicochemical, and microbial responses in a lassi-type beverage, with half-life values used to describe stability [39]. However, a kinetic half-life does not automatically correspond to loss of acceptability. Unless it is linked experimentally to product rejection, it should be interpreted as a measure of response stability, not as a validated estimate of shelf-life.

Different responses within the same product may also require different models. In yak-milk paneer, first-order and Arrhenius equations described temperature-dependent physicochemical changes, while modified Gompertz models described microbial growth [40]. This separation reflects the processes represented by each response: physicochemical trajectories indicate quality changes, whereas microbial acceptability depends on defined population limits. A good mathematical fit does not identify which response ultimately limits product life.

Temperature-dependent modeling adds another condition. The deterioration process observed under accelerated storage must remain relevant across the intended temperature range. Arrhenius and Q_10_ equations quantify the effect of temperature on deterioration rates, while Eyring and Ball models have also been applied to fermented dairy beverages [39]. Extrapolation becomes less reliable if temperature changes the dominant microbial population or biochemical pathway instead of only altering its rate. Models intended for distribution, retail, or domestic refrigeration should therefore be validated under realistic temperature histories.

Some fresh-dairy studies have progressed from constant to dynamic storage conditions. Microbial parameters estimated under isothermal conditions were evaluated using additional chhana data generated under controlled temperature fluctuations and excluded from the initial model fitting [41]. Models for *Listeria monocytogenes* and *Pseudomonas putida* in pasteurized milk have also incorporated temperature profiles measured in domestic refrigerators [43]. Such designs better represent practical storage conditions than experiments conducted exclusively at constant temperatures.

In predictive microbiology, primary and secondary models perform different functions. Primary models describe microbial populations over time. Modified Gompertz equations have been applied to standard plate counts, yeasts and molds, and coliforms in paneer [40]. Logistic, Gompertz, Baranyi, and three-phase linear models have been compared for microbial growth in chhana [41]. In pasteurized milk, biphasic growth at 4 °C was described with a biphasic Baranyi model, while monophasic Baranyi–Roberts models were used at higher temperatures [34].

Secondary models relate microbial growth parameters to environmental conditions. In paneer, an extended Ratkowsky square-root model described the growth rates of *L. monocytogenes*, while a separate model defined the growth/no-growth boundary as a function of temperature and pH [33]. Maximum growth rates in pasteurized milk have also been related to temperature using a Huang square-root model [34]. For chhana stored under fluctuating temperatures, microbial growth was described using a differential Baranyi model [41]. These approaches extend prediction beyond a single isothermal growth curve, but shelf-life estimation still requires a criterion for unacceptable microbial behavior.

The thresholds used across studies show why this criterion matters. Pasteurized milk shelf-life has been defined as the time required for total plate counts to reach a specified limit [34]. A concentration of 20,000 CFU/mL has also been adopted as a conservative quality threshold for spoilage caused by post-pasteurization contamination [14]. In probabilistic yogurt models, yeast populations above 5 log CFU/mL were classified as spoilage, although the relationship between this concentration and visible deterioration had not been established experimentally for the strains tested [23]. Model accuracy and endpoint validity must be assessed separately. Predicting when a population reaches a threshold does not confirm that the threshold corresponds to product rejection.

Stochastic models are appropriate when variability in contamination or storage conditions is part of the prediction problem. Monte Carlo simulation has been used to estimate consumer exposure to visible mold in yogurt, identifying the frequency of initial contamination as an important source of variation [22]. Probabilistic yogurt spoilage models have also incorporated differences in initial yeast contamination, species, temperature, growth rate, lag phase, and maximum population [23]. In fluid milk, post-pasteurization contamination was modeled probabilistically, and the resulting microbial distributions were compared with commercial validation data [14]. At day 7, the predicted and observed proportions of containers exceeding 20,000 CFU/mL were similar, providing evidence of performance outside the data used to calibrate the growth models.

Quality and safety can be examined within the same probabilistic framework, but their outcomes must remain separate. Models based on residential refrigerator temperature histories have evaluated both *P. putida* spoilage and *L. monocytogenes* behavior in pasteurized milk [43]. The same temperature profiles affected both organisms, but *P. putida* represented deterioration and *L. monocytogenes* represented microbiological risk. In a safety-focused application, log-linear, log-linear-with-shoulder, and Weibull models described the survival of *Escherichia coli* O157:H7 and *L. monocytogenes* in refrigerated kefir [42]. These models estimate pathogen persistence and do not represent sensory shelf-life.

Consumer rejection can be modeled probabilistically through survival analysis. This method estimates how the probability of rejection changes with storage time and accounts for differences among consumers in the time at which a product becomes unacceptable [44]. The selected rejection probability forms part of the operational shelf-life criterion and is specific to the study design and product conditions.

Uncertainty must also be considered independently of model fit. Biological variation, initial contamination, analytical error, parameter estimation, temperature fluctuations, and the choice of failure criterion can all affect the final shelf-life estimate. Monte Carlo simulations can represent several of these sources but cannot resolve uncertainty in the endpoint itself, as shown by the assumed yeast spoilage threshold used in yogurt modeling [23]. When sufficient data are available, prediction intervals or failure probabilities provide a fuller description of uncertainty than a single deterministic estimate.

The failure event should guide model selection. The deterioration or safety process determines the response of interest, the endpoint defines failure, and the observed trajectory determines the mathematical structure. The intended application then establishes the temperature range, validation design, and treatment of uncertainty. Analytical and data-driven methods can be assessed by how accurately they estimate the selected response and its proximity to the defined endpoint.

## 5. Emerging Analytical and Data-Driven Approaches

Analytical technologies complement conventional shelf-life assessment by translating chemical, physical, and microbiological changes into measurable signals. Their predictive value depends on the meaning of those signals. A sensor response, spectrum, image, or metabolomic profile may change consistently during storage without showing when the product becomes unacceptable. Its usefulness for shelf-life prediction depends on whether it is linked to the process and criterion that limit product life.

The selected studies provide different levels of predictive evidence. Some analytical responses mainly follow storage progression, such as UV–Vis models for yogurt age and metabolomic discrimination of storage periods [35,36]. Others estimate intermediate indicators, including microbial counts, acidity, and sensory scores [9,45,46]. Freshness classifiers assign samples to predefined quality categories based on acidity, sensory evaluation, storage stage, or related reference data [47,48,49]. Fewer studies connect the analytical signal to a criterion used to calculate time to failure. In one study, an electronic-tongue response was linked to both an aerobic plate count limit and a sensory discrimination threshold before shelf-life was extrapolated to 4 °C [50].

These outputs represent different prediction tasks. Storage-age models show that an analytical signal changes over time, while indicator models estimate a measurement associated with deterioration. Quality classifiers describe the current state of a product using predefined categories. Endpoint-based models go a step further by estimating when the product will reach a defined failure criterion. Table 2 classifies the analytical approaches used in the selected studies according to these prediction targets.

### 5.1. Analytical Technologies and Deterioration Signals

Sensor-based methods can monitor deterioration rapidly, but their outputs vary in how closely they reflect product failure. An electronic nose combined with artificial neural networks was used to estimate total bacterial counts in milk and classify samples into microbial-quality grades [9]. The model performed well with test and additional validation samples, but its target was bacterial concentration, not shelf-life. Such a system can contribute to shelf-life assessment when the predicted population is compared with an appropriate spoilage limit.

A voltammetric electronic tongue provided a more direct link between the sensor response and failure criteria [50]. The differential electrochemical response was modeled kinetically at accelerated storage temperatures. For microbiological assessment, the response was related to the time required for aerobic plate counts to exceed 50,000 CFU/mL, while sensory thresholds were established separately through triangle testing. Extrapolation to 4 °C produced relative errors of 13.57% for the microbiological estimate and 7.68% for the sensory estimate. Because the response was calibrated against defined reference criteria, the method provides evidence of endpoint-based shelf-life prediction.

Colorimetric sensors offer another option for rapid monitoring. A Schiff-reagent nanosensor detected volatile compounds produced during bacterial spoilage of pasteurized milk through a color change [51]. The color response correlated with aerobic plate counts at 13, 15, and 19 °C but was absent at 7 °C, even when bacterial populations reached approximately 6 log CFU/mL. Sensor performance was thus affected by storage temperature and the rate of volatile formation. A signal calibrated under accelerated conditions may not retain the same decision threshold under refrigeration.

A colorimetric sensor array combined with image analysis, a decision tree, and a smartphone application was also used to classify milk samples during storage [52]. The application achieved an overall error of approximately 3%, and samples were evaluated until total bacterial counts reached at least 7 log CFU/mL. The method classified storage or freshness status effectively but did not estimate the remaining time before a defined failure criterion was reached.

Electrical measurements have also been tested as indirect indicators of deterioration. Conductivity changes were converted into an electrical freshness index and related empirically to pH and microbial populations at 5 and 20 °C [45]. These relationships varied with temperature and microbial group. Lactic acid bacteria, for instance, could not be predicted from the index at 5 °C but were well described at 20 °C. Conductivity can thus indicate deterioration, although its interpretation depends on the microorganisms active under the storage conditions.

Packaging-integrated sensors allow quality to be assessed inside the package. A capacitive near-field communication (NFC) tag translated changes in the dielectric properties of milk into a binary fresh/spoiled result displayed on a smartphone [53]. A pH value of 6.3, obtained from the previous literature, was used to derive the critical dielectric constant. Milk source, packaging material, and temperature were recognized as possible causes of false-positive and false-negative results. The system is best interpreted as a threshold-based freshness classifier until its performance is validated across a broader range of products and conditions. A lac-dye indicator for packaged paneer changed color when microbial counts exceeded acceptable limits at 25 and 8 °C and supported PLS prediction of quality parameters, although no independent validation set was reported [55].

A bromocresol-purple optode provided a more extensive evaluation of intelligent packaging for milk acidification [47]. Principal Component Analysis (PCA), Linear Discriminant Analysis (LDA), and Partial Least Squares (PLS) were applied to the sensor data, and a titratable acidity value of 8 °SH separated fresh from spoiled samples. Models were tested with whole, semi-skimmed, and skimmed milk. The final classifier was evaluated with samples outside the original dataset, including sensors from another batch, and achieved 91.7% accuracy. This design provides evidence of transferability to new samples, although the endpoint was based on acidity and did not directly represent consumer rejection.

Spectroscopic methods generate broader analytical fingerprints but require the same attention to the prediction target. UV–Vis spectra were used with PLS to predict yogurt age during storage [35]. An external set of 16 spectra produced a standard error of prediction of approximately 3 days. The model confirmed that the spectral profile changed with storage time, but it predicted sample age and did not estimate when an independent quality limit would be reached.

Fourier-Transform Infrared Spectroscopy with Attenuated Total Reflectance (FTIR–ATR) spectra have been linked to conventional reference measurements using PLS and Support Vector Machine (SVM) models [46]. The models estimated total viable counts, lactic acid bacteria, lactococci/*Streptococcus thermophilus*, and sensory scores. Sensory evaluation classified samples as fresh, marginal, or unacceptable. One yogurt batch was used for model development and a second for validation. Predictions were generally successful for the main microbial groups and sensory status, while models for *Listeria monocytogenes* performed poorly. The same analytical platform may therefore perform differently for quality and safety targets.

Visible/near-infrared (Vis/NIR) and synchronous two-dimensional Vis/NIR spectroscopy have also been used to classify milk [48]. Fresh, sub-fresh, and spoiled samples were defined by titratable acidity ranges of 13–15, 15–18, and >18 °T, respectively. An SVM based on 2D-Vis/NIR spectra correctly classified all spoiled samples and 93.3% of sub-fresh samples in an additional set of 33 unknown samples. The use of physicochemical class boundaries provides stronger evidence of quality-state classification than categories based only on elapsed storage time.

Digital imaging can achieve similarly high classification accuracy; 34-layer Residual Neural Network (ResNet34) convolutional neural networks were used to monitor three yogurt formulations from one commercial brand [49]. Blind validation with images from batches excluded from model development produced accuracies of 96.95% and 96.86% under two acquisition conditions. This batch-level separation improved the evaluation design. However, the classes were based mainly on storage time and proximity to the best-before date. The model consequently classified freshness or storage stage but did not predict an independently established acceptability endpoint. Its restriction to one brand also limits transfer to other formulations and manufacturers.

Volatile and metabolomic methods provide detailed chemical information that can help identify deterioration pathways and candidate markers. In pasteurized milk, total microbial populations were associated with selected volatile ions and sensory attributes, although microbial numbers alone did not explain all sensory and volatile variation [18]. Marker responses may therefore depend on the composition and metabolic activity of the contaminating microbiota as well as on total population size.

Volatile organic compounds (VOCs), microbial load, and sensory changes have also been monitored during milk storage at different temperatures [54]. Associations among selected volatile compounds, bacterial populations, and proteolytic and lipolytic activities suggest that some VOCs may support chemical sensing of milk deterioration [15,51]. These relationships support their use as candidate markers. Their application to shelf-life prediction still requires a concentration or multivariate response linked to a defined failure criterion. A dual-channel fluorescence nanoprobe quantified biogenic amines in milk and yogurt and was also applied to time-dependent milk spoilage; however, no product-specific acceptability threshold or time-to-failure model was established [56].

Metabolomic studies have mainly characterized storage-related chemical changes. Nuclear Magnetic Resonance (NMR) analysis identified organic acids and other microbial metabolites that varied with milk storage time and temperature [30]. The aim was to identify candidate spoilage markers, without defining an acceptability threshold. Ultra-High-Performance Liquid Chromatography coupled with Quadrupole Time-of-Flight Mass Spectrometry (UHPLC–QTOF–MS) and Orthogonal Partial Least Squares–Discriminant Analysis (OPLS–DA) have also been used to characterize chemical changes in ricotta, with storage time used as the class variable [36]. Although the model separated storage periods, the chemical profile was not translated into a rejection criterion.

NMR profiles of mozzarella stored under adequate and irregular refrigeration provided a more operational estimate of chemical deterioration [31]. Conventional compositional and physicochemical measurements contributed little to group separation, while metabolites, especially succinate, distinguished the refrigeration histories. Concentrations measured under inadequate refrigeration were compared with those found at the labeled expiry date under adequate refrigeration to estimate the loss of chemical shelf-life. This approach detected accelerated deterioration, but the reference remained the labeled expiry condition and was not based on an independently determined sensory or microbiological endpoint.

The analytical platform is only one part of the prediction system. Sensors, spectroscopy, imaging, and metabolomics can describe the current state of a product. Their contribution to shelf-life prediction is stronger when the measured response is linked to a defined quality or safety criterion, evaluated with samples excluded from model development, and tested under the intended storage conditions.

### 5.2. Chemometrics and Machine Learning

Analytical datasets from fresh dairy products range from individual physicochemical measurements to spectral, imaging, metabolomic, and sensor-array profiles containing hundreds or thousands of variables. Chemometric and machine learning methods are used to explore these data, select variables, estimate continuous responses, and classify samples. The selected studies applied PCA, three-way PCA, partial least squares (PLS) regression, OPLS–DA, SVM, artificial neural networks (ANN), genetic algorithms (GA), decision trees, and convolutional neural networks (CNN).

The prediction target matters more than the choice of algorithm. Unsupervised methods such as PCA, three-way PCA, and principal response curves can identify storage trajectories, refrigeration effects, and candidate markers, but they do not independently predict shelf-life [30,31,36]. Supervised models address specific targets. PLS, with or without GA-based wavelength selection, has been used to estimate yogurt storage age from UV–Vis spectra [35]. ANN models have predicted total bacterial counts and microbial-quality grades in milk [9]. PLS and SVM models have estimated microbial populations, sensory scores, and sensory-quality classes [46]. Other classifiers have assigned milk and yogurt samples to predefined freshness or storage states [48,49].

These applications represent distinct prediction tasks. Estimating storage age confirms that the analytical response follows changes over time but does not identify the point of product rejection. Predicting a microbial population can support shelf-life assessment if the result is compared with a justified spoilage or safety limit. Classification into fresh, marginal, or unacceptable states is more closely related to product quality when the class boundaries come from independent sensory, microbiological, or physicochemical criteria. Classes based mainly on storage time or a labeled best-before date describe storage status, not an experimentally determined failure endpoint.

The selected studies demonstrate these differences. A model for yogurt age produced a standard error of prediction of approximately 3.3 days with a test set of 16 spectra, but the dependent variable was storage time [35]. Another study reported close agreement between predicted and measured bacterial counts and evaluated the ANN with 24 additional samples excluded from training and initial testing [9]. Even with this stronger evaluation, the model predicted bacterial counts and not time to product failure. In a separate study, one yogurt batch was used for model development and another for validation [46]. The spectral models performed well for several microbial groups and sensory responses, but predictions for *Listeria monocytogenes* were much weaker. Performance for quality-related responses cannot be assumed to apply to a safety target measured with the same platform.

Freshness-classification studies also differ in how their target classes are defined. Milk samples classified as fresh, sub-fresh, or spoiled according to titratable acidity were evaluated with 33 additional samples [48]. The 2D-Vis/NIR SVM correctly classified all spoiled samples and 93.3% of sub-fresh samples. CNN-based yogurt classification achieved blind-validation accuracies of approximately 97% with images from batches excluded from model development [49]. However, the classes were based mainly on storage progression relative to the best-before date, and all formulations came from one commercial brand. The high accuracy shows that the model recognized the specified storage classes, but it does not establish direct prediction of an independent acceptability endpoint.

Model complexity does not provide evidence of better shelf-life prediction by itself. Nonlinear methods such as SVMs, ANNs, and CNNs may outperform simpler models in a given dataset, but predictive performance refers to accuracy in new observations, not fit to the development data [57]. Algorithms can be compared fairly only when they use the same target, samples, preprocessing, data partition, and performance metric. Differences in reported accuracy may otherwise arise from the experimental design or target definition.

Dependence among observations is especially important in dairy datasets containing repeated spectra, images, storage times, or measurements from the same production batch. Random allocation of closely related observations to both training and test sets may produce overly optimistic results because the two subsets retain characteristics of the same experimental unit [58]. Leakage can also occur when variable selection, preprocessing, dimensionality reduction, or hyperparameter tuning uses information from evaluation samples. When cross-validation supports both model selection and final performance estimation, nested cross-validation or an untouched test set provides a more reliable estimate of predictive error [59].

One of the clearest endpoint-based applications connected an electronic-tongue response separately to an aerobic plate count limit and a sensory discrimination threshold [50]. The response was then incorporated into kinetic and Arrhenius models to estimate shelf-life. Most other chemometric and machine learning studies predicted storage age, current quality state, microbial or sensory indicators, or candidate markers. These remain useful tasks, but they should be named according to their actual output.

Machine learning connects analytical information to a specified prediction target. Its value in shelf-life assessment depends on the relevance of that target, the quality and independence of the reference data, and the conditions represented during model development. A more complex algorithm cannot correct an unclear endpoint or a reference variable with only an indirect relationship to product failure.

### 5.3. Validation, Transferability, Data Integration, and Application to Shelf-Life

Validation should match the intended use of the model. Agreement with the development data measures fit, while predictions for observations excluded from model construction provide evidence of performance [60,61]. The strength of this evidence depends on the independence of the evaluation samples.

The selected studies used several evaluation designs. Cross-validation reuses observations from the development dataset and supports model selection and internal assessment. A hold-out test set provides a stronger evaluation when it remains untouched throughout training and model selection. Holding out an entire production batch also tests biological and manufacturing variation. Evaluation across brands, formulations, production periods, sites, instruments, or storage scenarios examines broader transferability. These designs represent different levels of generalization and should be reported accordingly.

Data partitioning must reflect the model’s intended application. Spectra, images, storage times, and technical replicates obtained from the same product or batch are not fully independent. Randomly distributing them between training and evaluation sets can underestimate prediction error because both subsets contain information from the same experimental unit [58]. Models intended for new production batches should be evaluated with entire batches excluded from development. The same principle applies when transfer across brands, formulations, manufacturing sites, or temperature histories is claimed.

Data processing must preserve this separation. Scaling parameters, feature or wavelength selection, dimensionality reduction, and hyperparameter optimization should be estimated from the training data and then applied to the evaluation set. Processing the full dataset before partitioning allows evaluation samples to influence model construction. The same cross-validation results should also not be used both to select a model and to report its final performance, as this may underestimate prediction error [59]. Nested cross-validation or an untouched evaluation set separates model selection from final assessment.

Validation designs varied considerably across the selected dairy studies. An external set of 16 randomly selected spectra was used to test yogurt storage-age prediction [35]. This design assessed hold-out performance within the same experiment but did not test a new production batch or formulation. An ANN was later evaluated with 24 additional milk samples that had not been used for training or initial testing, although transfer across production settings was not examined [9]. Stronger batch-level evidence came from a study that developed the model with one yogurt batch and validated it with another [46].

Transfer outside the original calibration set has also been examined directly. Sensor models were first evaluated within individual milk types, then across whole, semi-skimmed, and skimmed milk, and finally with unknown samples outside the original dataset [47]. The final LDA model achieved 91.7% accuracy. Sensors from another production batch were included, allowing sensor-to-sensor variation to form part of the evaluation. This feature increased the practical relevance of the design for intelligent packaging.

A blind-validation set containing images from yogurt batches excluded from model development provided a stronger test than random partitioning at the image level [49]. All samples still came from the same commercial brand, which limits generalization to other formulations and manufacturers. Independence at the batch level does not establish unrestricted transferability across products.

Model validation and endpoint validation address separate questions. A model may reproduce its reference variable accurately even when the criterion used to define failure remains uncertain. For example, a colorimetric VOC sensor tracked microbial spoilage at 13–19 °C but did not produce the same response at 7 °C, despite substantial bacterial growth [51]. The relationship between sensor response and deterioration changed with temperature. In another study, a fresh/spoiled classifier used a pH threshold taken from the literature, while milk source, packaging material, and temperature were recognized as possible influences on sensor response [53]. Both the analytical signal and its linked criterion require evaluation across the intended conditions of use.

Temperature history requires the same attention. Models developed under constant temperatures may describe deterioration within the experimental range but perform differently during the fluctuations encountered in distribution, retail, and domestic storage. Microbial models have been evaluated with additional chhana data generated under fluctuating temperatures [41]. Measured temperature histories from domestic refrigerators have also been incorporated into model evaluation [43]. Comparable testing under realistic time–temperature conditions is still uncommon in analytical and machine learning studies.

Data integration may improve prediction when each measurement describes a different component of deterioration. Microbial populations, volatile compounds, and sensory attributes have been examined together in pasteurized milk, revealing related but non-identical patterns [18]. FTIR spectra have been linked to microbial and sensory reference measurements [46], and one electronic-tongue response has been calibrated separately against microbiological and sensory criteria [50]. Combining measurements is useful when each source contributes information relevant to product failure, not when it only adds correlated variables [62].

The benefit of additional data sources should be confirmed in independent samples. A complex multimodal model may fit the development dataset more closely without transferring better to new products or conditions. Biological and physicochemical interpretation of the selected variables can help determine whether the relationship is likely to remain stable after changes in batch composition, microbial ecology, or storage conditions.

Uncertainty should be included in the final shelf-life estimate. Initial product quality, microbial contamination, model parameters, analytical error, temperature history, and the selected failure criterion can all affect the predicted time to failure. A single estimate without an uncertainty measure may suggest more precision than the data support. Prediction intervals, confidence limits, and probabilities of exceeding the failure criterion provide more useful information when they can be estimated reliably [61,63].

Validation of fresh-dairy shelf-life models involves three questions: Is the analytical signal reproducible? Does the model predict its reference response in genuinely new samples? Does that response remain linked to the relevant quality or safety endpoint under the intended conditions of use? A model developed for quality monitoring can be considered a transferable shelf-life prediction system only when all three conditions are supported by evidence.

## 6. Research Gaps and Limitations

A recurring limitation in the literature is the gap between monitoring deterioration and predicting the end of shelf-life. Many studies detect storage-related changes or estimate intermediate responses such as microbial counts, acidity, sensory scores, and storage age. These measurements support quality assessment but contribute directly to shelf-life prediction only when they are linked to a defined failure criterion [12,64]. Some studies have identified clear analytical changes during storage without establishing an independent acceptability endpoint [35,36]. Others have estimated shelf-life by linking the analytical response to microbiological and sensory criteria [50].

Endpoint definition remains a major methodological gap. Microbial populations, acidity, texture, volatile compounds, metabolite profiles, and sensory attributes can change at different rates. The factor that limits product life may also vary with formulation, batch, packaging, and storage conditions. Studies that evaluated several criteria in ricotta and yogurt found that shelf-life was determined by the first limit reached, not by the variable with the largest analytical change [3,25]. Quality and safety endpoints must also be kept separate. Models of spoilage or sensory rejection describe different outcomes from models of pathogen growth, growth/no-growth boundaries, or survival [33,42].

Validation is another limitation. Strong classification or regression results within the development dataset do not guarantee similar performance in new production batches or under different processing and storage conditions. The selected studies used evaluation designs ranging from internal cross-validation and randomly selected hold-out samples to independent samples and entire production batches. Batch-level validation is especially important because raw-material composition, initial microbiota, formulation, processing, packaging, and analytical response can vary between production runs. The validation design must match the level of generalization claimed for the model [58,59].

Transferability depends on the range of conditions included during evaluation. A colorimetric response associated with milk spoilage at elevated temperatures was not reproduced at 7 °C [51]. Image-based yogurt classification achieved high blind-validation accuracy, but the proof-of-concept dataset included only one commercial brand [49]. By comparison, some sensor models were tested across several milk types and with an additional independent dataset [47]. These differences in experimental scope determine how far a model can be applied beyond its development conditions.

The link between the analytical signal and the endpoint also needs validation. A model may predict storage time, microbial concentration, acidity, or freshness class accurately while the relationship between that output and product rejection remains uncertain. This issue is particularly relevant to metabolomic, spectroscopic, and sensor-based methods, which can identify many variables that change during storage. Before being described as shelf-life markers, candidate variables must be tested against the limiting deterioration process and an explicit microbiological, physicochemical, structural, or sensory criterion.

Realistic storage histories are also poorly represented. Constant-temperature experiments are useful for kinetic characterization, but commercial products encounter temperature fluctuations during distribution, retail, and domestic storage. Dynamic temperature experiments better reflect these conditions [41,43]. Similar testing is needed for analytical and machine learning systems intended for use throughout the cold chain.

Most models estimate shelf-life from the initial condition of the product. Industrial applications would benefit from systems that update the remaining shelf-life as new sensor readings, analytical measurements, or temperature records become available [64]. Developing these systems requires a validated deterioration model, an estimate of the current product state, and information about its previous storage history.

Uncertainty is seldom included explicitly in the final prediction. Biological variation, initial contamination, analytical error, parameter estimation, temperature fluctuations, and the choice of failure criterion can all affect the estimated time to product failure. Reporting a single value may imply more precision than the data support. When sufficient data are available, prediction intervals, confidence limits, or endpoint probabilities provide a more realistic description of shelf-life uncertainty [61,63].

Data integration may improve prediction when the measurements describe different deterioration processes. Adding more variables alone offers no guarantee of better performance. Microbial, volatile, physicochemical, spectral, and sensory responses may evolve at different rates, while highly correlated measurements can increase model complexity without adding useful information. Integrated models must show that each data source contributes information related to the endpoint and improves prediction in genuinely independent samples [62].

Further work requires experimentally justified endpoints, representative multi-batch datasets, validation at the intended level of application, realistic time–temperature histories, and explicit treatment of uncertainty. These elements are more important for practical shelf-life prediction than additional analytical variables or more complex algorithms.

## 7. Conclusions

Reliable shelf-life assessment in fresh dairy products requires more than the detection of storage-related changes. Predictive relevance depends on identifying the process that limits product quality or safety, selecting a representative response, and defining the corresponding failure criterion before model development. Kinetic and probabilistic models, analytical technologies, chemometrics, and machine learning methods can then be evaluated according to how accurately they estimate that response and the time required to reach the endpoint.

Current evidence shows a clear distinction between quality monitoring and endpoint-based shelf-life prediction. Storage-age discrimination, candidate-marker identification, microbial-count prediction, or high classification accuracy can provide useful information on product status, but none alone establishes when a product becomes unacceptable. Stronger evidence is obtained when analytical or model-derived responses are evaluated against explicit microbiological, physicochemical, structural, or sensory criteria using independent samples.

Rapid sensor, spectroscopic, and imaging technologies are particularly suited to routine and potentially in-line or in-package quality assessment, provided that their signals remain linked to the relevant endpoint across batches and storage conditions. Metabolomic and volatile profiling offer complementary value for identifying deterioration pathways and candidate markers, but their translation into operational shelf-life tools requires validated thresholds and simpler measurement strategies.

For industrial application, models need representative multi-batch data, validation under realistic time–temperature conditions, a clearly defined domain of application, and explicit treatment of prediction uncertainty. Greater attention to these requirements, together with assessment of transferability across products and production conditions, is needed to move from storage monitoring toward reliable and updateable shelf-life prediction in fresh dairy products.

## Figures and Tables

**Figure 1:**
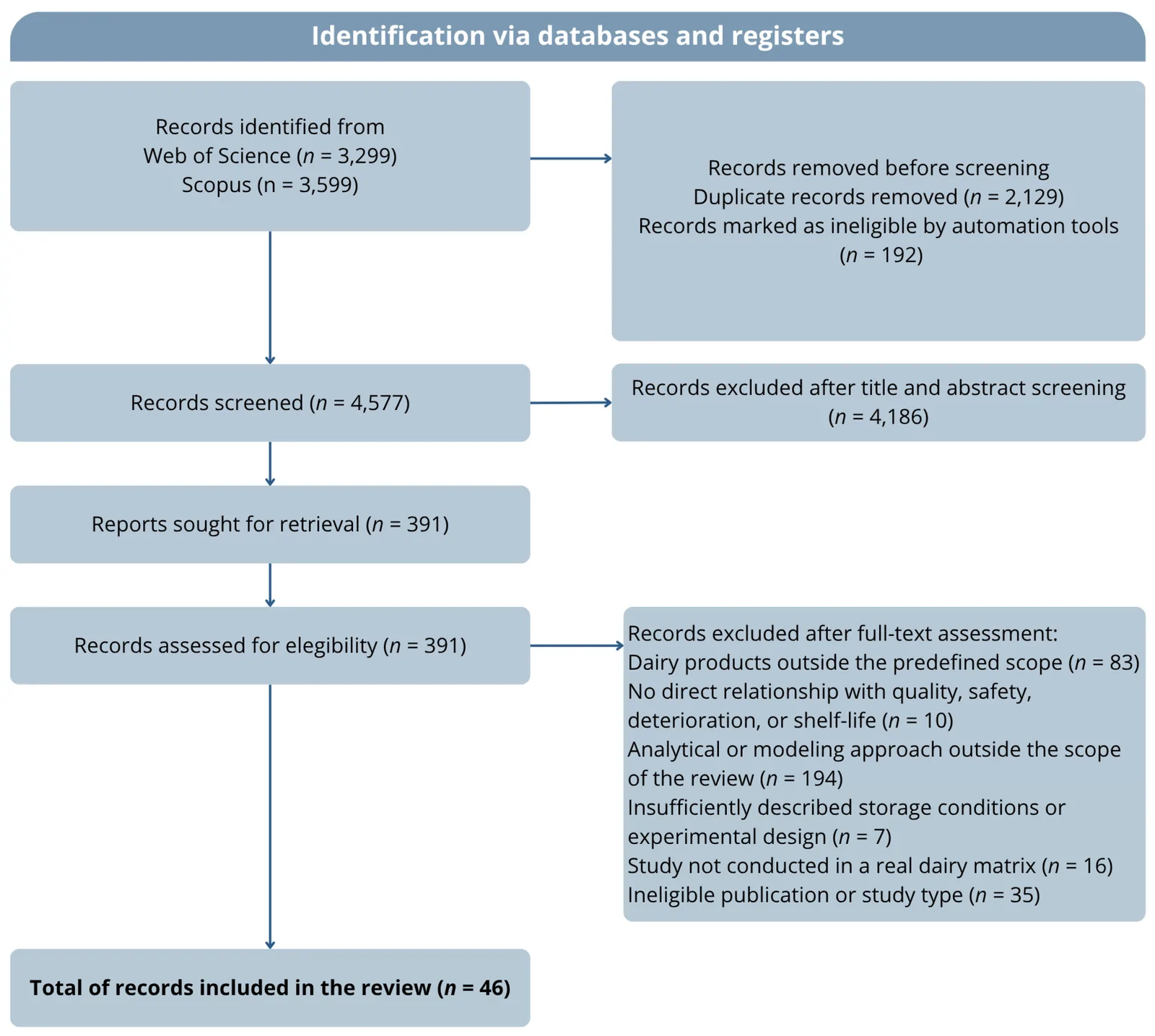
Flow diagram of study identification, screening, eligibility assessment, and inclusion in the critical narrative review.

**Figure 2:**
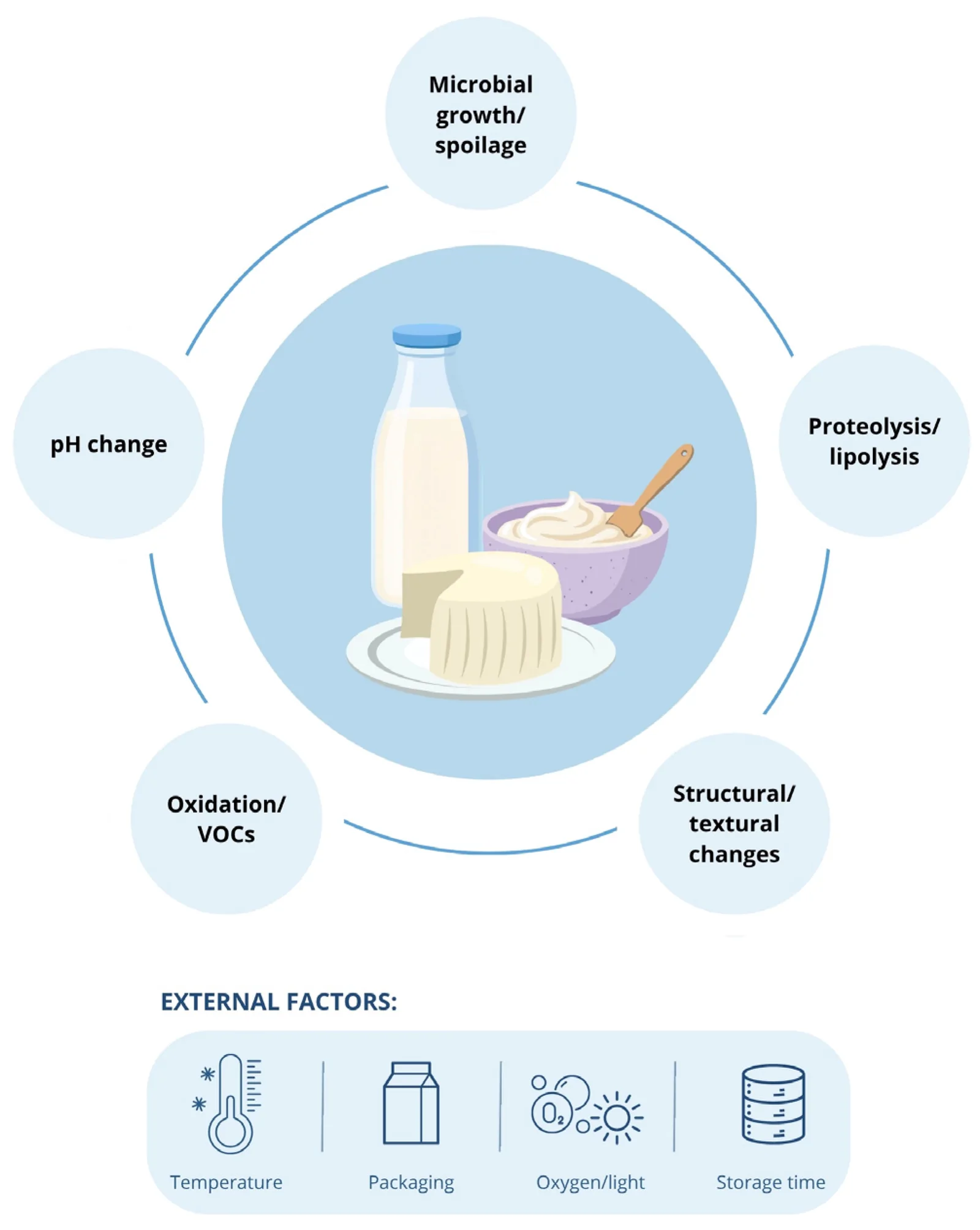
Main deterioration pathways in fresh refrigerated dairy products and the external factors influencing their progression. VOCs, volatile organic compounds.

**Figure 3:**
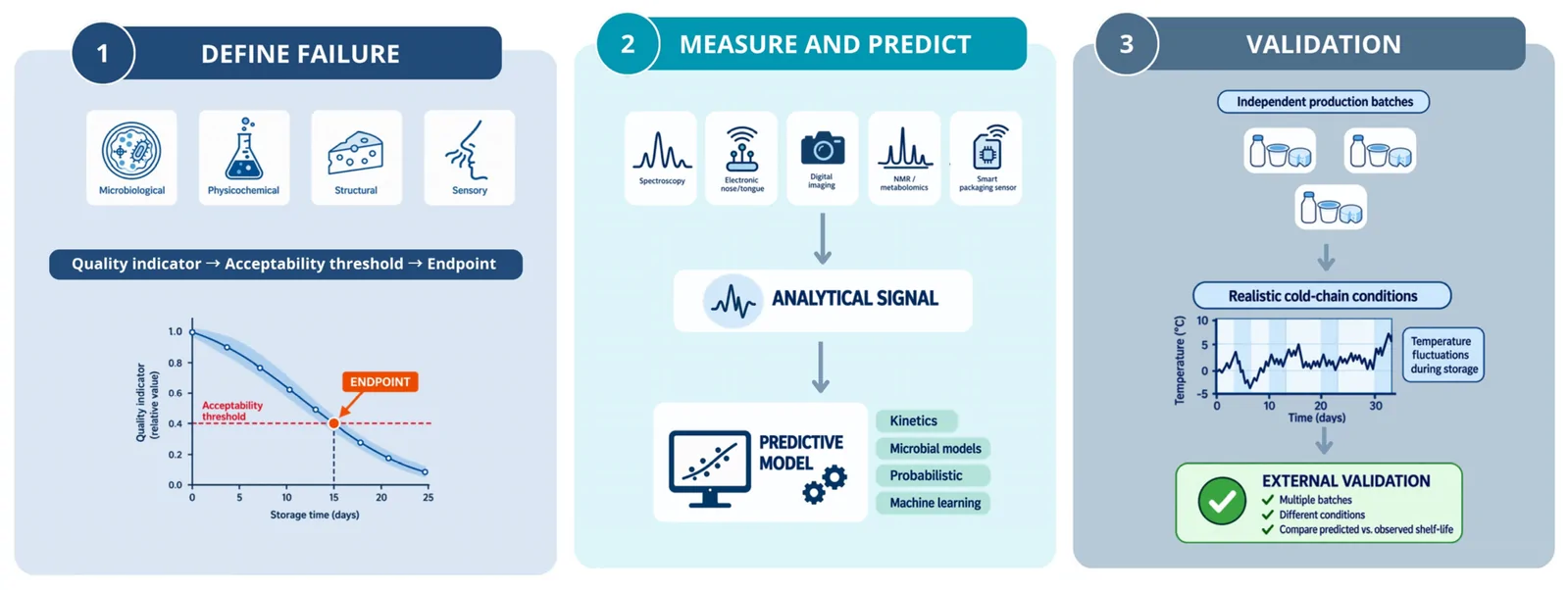
Conceptual framework for endpoint-based shelf-life prediction in fresh dairy products, covering failure definition, analytical measurement and modeling, and external validation.

**Table 1:** Kinetic and probabilistic models applied to quality, shelf-life, and safety assessment in fresh dairy products.

Modeling Approach	Model	Response Modeled	Endpoint or Criterion Used	Dairy Product	Reference
Reaction order/temperature-dependence kinetics	First-order kinetics and Arrhenius	Acidity, viscosity, and sensory score	Quality-related endpoints: predefined acceptability limits for acidity, viscosity, and sensory score. Overall shelf-life was defined by the earliest limit reached. At 5 °C, predicted times for one yogurt were 16.1, 20.3, and 15.5 d, respectively.	Yogurt	[3]
Reaction order/temperature-dependence kinetics	First-order degradation; zero-order color kinetics; Arrhenius/Q_10_	Betalain concentration and color parameters	Quality-related attribute stability; no product-rejection endpoint: betalain half-life and color-change kinetics described individual quality attributes without defining the end of acceptable yogurt shelf-life.	Betalain-enriched yogurt	[38]
Reaction order/temperature-dependence kinetics	Zero-, first-, and second-order kinetics and Arrhenius	Overall acceptability, acidity, FFA, TBA, total plate count, and yeasts/molds	Quality-related surrogate endpoint: kinetic half-life (t_1/2_) was used as a shelf-life estimate for the measured responses, but its correspondence with product rejection was not independently validated.	Lassi-type dairy beverage	[39]
Reaction order kinetics/primary microbial growth	First-order and Arrhenius; modified Gompertz	Moisture, pH, titratable acidity, FFA, tyrosine value; SPC, yeasts/molds, and coliforms	Quality-related responses and microbiological acceptability criteria: physicochemical changes were modeled kinetically, while microbial counts were assessed against BIS/FSSR limits. No single integrated product-failure endpoint was defined.	Vacuum-packed yak-milk paneer	[40]
Primary microbial growth	Three-phase linear growth model	TPC, coliforms, yeasts and molds	Quality-related microbial endpoints: critical concentrations were 5.544 log CFU/g for TPC, 2 log CFU/g for coliforms, and 2.176 log CFU/g for yeasts/molds. TPC reached its limit first and therefore determined the estimated shelf-life under the conditions studied.	Chhana	[41]
Primary and secondary microbial growth	Biphasic/monophasic Baranyi–Roberts and Huang square-root	Total plate count and temperature-dependent growth rate	Quality-related microbial endpoint: shelf-life was defined as the time required for TPC to exceed 4.47 log CFU/mL, using the FSSAI criterion reported in the study.	Pasteurized milk	[34]
Primary microbial growth/sensory deterioration	Re-parameterized Gompertz models	*Pseudomonas* spp., yeasts, and overall sensory quality	Quality-related microbial and sensory endpoints: limits were 7 log CFU/g for *Pseudomonas* spp. and yeasts, and an overall sensory-quality score of 4/7. Shelf-life was defined by the first acceptability limit reached.	Ricotta	[25]
Secondary/growth-boundary microbial model	Extended Ratkowsky square-root and logistic growth/no-growth model	*Listeria monocytogenes* growth rate and growth/no-growth behavior	Safety-related growth criterion: an increase of ≥1 log_10_ CFU/mL was used to distinguish growth from no growth of *L. monocytogenes.* This criterion describes pathogen behavior, not sensory rejection or a demonstrated safety limit for consumption.	Paneer	[33]
Microbial survival	Log-linear, log-linear with shoulder, and Weibull	Survival/inactivation of *E. coli* O157:H7 and *L. monocytogenes*	Safety-related outcome; no explicit product-failure endpoint: models described survival/inactivation of *E. coli* O157:H7 and *L. monocytogenes*, without defining a sensory or commercial shelf-life endpoint.	Kefir	[42]
Stochastic/probabilistic microbial model	Monte Carlo simulation	Distribution of post-pasteurization contaminant populations and probability of spoilage	Quality-related spoilage endpoint: an SPC of 20,000 CFU/mL was used to define spoilage. Predicted microbial outcomes were evaluated against commercial data at days 7 and 10.	Pasteurized milk	[14]
Stochastic/probabilistic microbial model	Monte Carlo simulation	Yeast population and predicted proportion of spoiled cartons	Quality-related assumed spoilage endpoint: yeast populations exceeding 5 log_10_ CFU/mL were assumed to indicate visible spoilage. *	Yogurt	[23]

Note: FFA, free fatty acids; FSSR, Food Safety and Standards Regulations; FSSAI, Food Safety and Standards Authority of India; SPC, standard plate count; TBA, thiobarbituric acid response; TPC, total plate count. * The exact detectable spoilage level was not experimentally determined for the four strains, which the authors identified as a source of uncertainty.

**Table 2:** Analytical and data-driven approaches used for storage monitoring, quality classification, and endpoint-related assessment in fresh dairy products.

Analytical Approach	Product	Model Output	Endpoint or Reference Criterion	Interpretation for Shelf-Life Assessment	Reference
Electronic nose and ANN	Pasteurized milk	Total bacterial count and microbial-quality grade	TBC-based microbial grades; no independent product-rejection endpoint	Predicts microbial status, not time to product failure	[9]
Voltammetric electronic tongue and kinetic/Arrhenius modeling	Pasteurized milk	Microbiological-indicator and sensory shelf-life estimates	APC limit of 50,000 CFU/mL; sensory discrimination threshold from triangle testing	Direct analytical-signal-to-criterion shelf-life estimation	[50]
Schiff-reagent colorimetric nanosensor	Pasteurized milk	VOC-related color change associated with bacterial growth	Color response compared with APCs in the spoilage range; response ineffective at 7 °C	Potential spoilage indicator, but temperature-dependent and not consistently applicable under refrigeration	[51]
Colorimetric optoelectronic nose and smartphone/decision tree	Milk	Storage/freshness classification	Samples monitored until TBC ≥7 log CFU/mL	Freshness-stage classification; does not directly estimate time to a validated failure endpoint	[52]
Electrical conductivity freshness index	Cow milk	Estimated pH and microbial populations	pH and TAB/PCB/LAB used as reference responses; no single product-level rejection criterion	Predicts deterioration indicators; performance depends on temperature and microbial group	[45]
Capacitive NFC sensor	Packaged milk	Fresh/spoiled classification	pH 6.3 adopted as the critical value for spoilage	Binary classification based on an assumed physicochemical threshold; affected by milk source, packaging, and temperature	[53]
BCP-EVOH optode and LDA/PLS	Milk	Fresh/spoiled class and titratable-acidity prediction	8 °SH used as the delimiter between fresh and spoiled milk	Endpoint-related acidity classification with independent test samples; does not estimate remaining shelf-life directly	[47]
UV–Vis spectroscopy and PLS	Yogurt	Storage age	Storage time; external test set used for prediction	Storage-age prediction rather than acceptability-endpoint prediction	[35]
FTIR–ATR and PLS/SVM	Yogurt	Microbial counts, sensory scores, and fresh/marginal/unacceptable classes	Sensory classes defined from panel scores; second batch used for external evaluation	Strong evidence for current quality-state assessment; not a direct time-to-failure model	[46]
2D-Vis/NIR and LDA/SVM	Milk	Fresh, sub-fresh, and spoiled classes	Acidity classes: 13–15 °T, 15–18 °T, and >18 °T, respectively	Quality classification against an explicit physicochemical criterion; unknown samples used for evaluation	[48]
Digital imaging and CNN	Yogurt	Freshness/storage-stage classification	Classes defined primarily relative to storage time and best-before date	High blind-test accuracy, but the target is storage/best-before status rather than an independently validated rejection endpoint	[49]
PTR-MS/VOC profiling	Pasteurized milk	Association among VOCs, microbial load, and sensory attributes	No single failure criterion	Characterizes deterioration relationships and potential indicators; does not directly estimate shelf-life	[18]
NMR metabolomics	Pasteurized milk	Storage- and temperature-responsive metabolites	Storage time and temperature; no rejection threshold	Candidate-marker identification and deterioration monitoring	[30]
NMR and multivariate analysis	Mozzarella	Chemical changes associated with adequate and inadequate refrigeration	Marker concentrations compared with those observed at the labelled expiry date under adequate refrigeration	Estimates chemical deterioration relative to a reference storage condition rather than a sensory or microbiological endpoint	[31]
UHPLC–QTOF–MS metabolomics and OPLS–DA	Ricotta	Discrimination among storage time points	Storage period used as class membership criterion	Storage-related chemical discrimination; no explicit rejection endpoint	[36]
VOC analysis and microbiological/sensory measurements	Pasteurized milk	Deterioration-associated volatile patterns	Microbial load and sensory quality monitored in parallel	Supports identification of spoilage-related markers but does not provide direct endpoint prediction	[54]

Note: ANN, artificial neural network; APC, aerobic plate count; BCP, bromocresol purple; FTIR–ATR, Fourier-transform infrared spectroscopy with attenuated total reflectance; LDA, linear discriminant analysis; NFC, near-field communication; NMR, nuclear magnetic resonance; OPLS–DA, orthogonal partial least squares–discriminant analysis; PLS, partial least squares; PTR-MS, proton-transfer-reaction mass spectrometry; SVM, support vector machine; TAB, total aerobic bacteria; TBC, total bacterial count; UHPLC–QTOF–MS, ultra-high-performance liquid chromatography coupled with quadrupole time-of-flight mass spectrometry; Vis/NIR, visible/near-infrared spectroscopy; VOC, volatile organic compound.

## Data Availability

No new data were created or analyzed in this study. Data sharing is not applicable to this article.

## Supplementary Materials

The following supporting information can be downloaded at https://www.mdpi.com/article/10.3390/foods15193439/s1: Table S1: Database-specific search strategy and Boolean search strings used to identify studies on shelf-life assessment and prediction in fresh dairy products; Table S2: Evidence map of selected studies on shelf-life assessment and prediction in fresh dairy products, including analytical approaches, modeling methods, endpoints, validation, and predictive performance; Table S3: SANRA self-assessment of the revised narrative review.

## Abbreviations

CFU	Colony-forming units
FTIR–ATR	Fourier-transform infrared spectroscopy with attenuated total reflectance
NFC	Near-field communication
NMR	Nuclear magnetic resonance
OPLS–DA	Orthogonal partial least squares–discriminant analysis
PCA	Principal component analysis
PLS	Partial least squares
PTR–MS	Proton-transfer-reaction mass spectrometry
SANRA	Scale for the Assessment of Narrative Review Articles
UHPLC–QTOF–MS	Ultra-high-performance liquid chromatography coupled with quadrupole Time-of-flight mass spectrometry
UV–Vis	Ultraviolet–visible spectroscopy
Vis/NIR	Visible and near-infrared spectroscopy

## References

[1] Fan X., Wang C., Cheng M., Wei H., Gao X., Ma M., Wang X., Li Z. (2023). Markers and Mechanisms of Deterioration Reactions in Dairy Products. Food Eng. Rev..

[2] Martin N.H., Torres-Frenzel P., Wiedmann M. (2021). Invited Review: Controlling Dairy Product Spoilage to Reduce Food Loss and Waste. J. Dairy Sci..

[3] Zhi N.-N., Zong K., Thakur K., Qu J., Shi J.-J., Yang J.-L., Yao J., Wei Z.-J. (2018). Development of a Dynamic Prediction Model for Shelf-Life Evaluation of Yogurt by Using Physicochemical, Microbiological and Sensory Parameters. CyTA-J. Food.

[4] Hayes E., Greene D., O’Donnell C., O’Shea N., Fenelon M.A. (2023). Spectroscopic Technologies and Data Fusion: Applications for the Dairy Industry. Front. Nutr..

[5] Lei T., Sun D.-W. (2019). Developments of Nondestructive Techniques for Evaluating Quality Attributes of Cheeses: A Review. Trends Food Sci. Technol..

[6] Pu Y.-Y., O’Donnell C., Tobin J.T., O’Shea N. (2020). Review of Near-Infrared Spectroscopy as a Process Analytical Technology for Real-Time Product Monitoring in Dairy Processing. Int. Dairy J..

[7] Freire P., Freire D., Licon C.C. (2025). A Comprehensive Review of Machine Learning and Its Application to Dairy Products. Crit. Rev. Food Sci. Nutr..

[8] Gupta M.K., Viejo C.G., Fuentes S., Torrico D.D., Saturno P.C., Gras S.L., Dunshea F.R., Cottrell J.J. (2022). Digital Technologies to Assess Yoghurt Quality Traits and Consumers Acceptability. J. Sci. Food Agric..

[9] Yang Y., Wei L. (2021). Application of E-Nose Technology Combined with Artificial Neural Network to Predict Total Bacterial Count in Milk. J. Dairy Sci..

[10] Rocha R.S., Silva R., Guimarães J.T., Balthazar C.F., Pimentel T.C., Neto R.P.C., Tavares M.I.B., Esmerino E.A., Freitas M.Q., Cappato L.P. (2020). Possibilities for Using Ohmic Heating in Minas Frescal Cheese Production. Food Res. Int..

[11] Rashvand M., Ren Y., Sun D.-W., Senge J., Krupitzer C., Fadiji T., Miró M.S., Shenfield A., Watson N.J., Zhang H. (2025). Artificial Intelligence for Prediction of Shelf-Life of Various Food Products: Recent Advances and Ongoing Challenges. Trends Food Sci. Technol..

[12] Andrewes P. (2022). Predicting the Shelf-Life of Microbially-Stabilised Dairy Products: What Are the Roles of Stability Studies, Storage Trials, ‘Accelerated’ Trials, and Dairy Science?. Int. Dairy J..

[13] Baethge C., Goldbeck-Wood S., Mertens S. (2019). SANRA—A Scale for the Quality Assessment of Narrative Review Articles. Res. Integr. Peer Rev..

[14] Lau S., Trmcic A., Martin N.H., Wiedmann M., Murphy S.I. (2022). Development of a Monte Carlo Simulation Model to Predict Pasteurized Fluid Milk Spoilage Due to Post-Pasteurization Contamination with Gram-Negative Bacteria. J. Dairy Sci..

[15] Ziyaina M., Govindan B.N., Rasco B., Coffey T., Sablani S.S. (2018). Monitoring Shelf Life of Pasteurized Whole Milk Under Refrigerated Storage Conditions: Predictive Models for Quality Loss. J. Food Sci..

[16] Glantz M., Rosenlöw M., Lindmark-Månsson H., Buhelt Johansen L., Hartmann J., Höjer A., Waak E., Löfgren R., Hallin Saedén K., Svensson C. (2020). Impact of Protease and Lipase Activities on Quality of Swedish Raw Milk. Int. Dairy J..

[17] El Dessouky Abdel-Aziz M., Darwish M.S., Mohamed A.H., El-Khateeb A.Y., Hamed S.E. (2020). Potential Activity of Aqueous Fig Leaves Extract, Olive Leaves Extract and Their Mixture as Natural Preservatives to Extend the Shelf Life of Pasteurized Buffalo Milk. Foods.

[18] Alothman M., Lusk K.A., Silcock P.J., Bremer P.J. (2018). Relationship between Total Microbial Numbers, Volatile Organic Compound Composition, and the Sensory Characteristics of Whole Fresh Chilled Pasteurized Milk. Food Packag. Shelf Life.

[19] Ziyaina M., Rasco B., Coffey T., Mattinson D.S., Sablani S. (2019). Correlation of Volatile Compound Concentrations with Bacterial Counts in Whole Pasteurised Milk under Various Storage Conditions. Int. J. Dairy Technol..

[20] Pereira F.A.B., Luiz L.L., Bruzaroski S.R., Poli-Frederico R.C., Fagnani R., Santana E.H.W. (2019). The Effect of Cold Storage, Time and the Population of *Pseudomonas* Species on Milk Lipolysis. Grasas Aceites.

[21] Paulo Vieira C., Pereira da Costa M., da Silva Frasão B., de Melo Silva V.L., Vilela de Barros Pinto Moreira R., Chifarelli de Oliveira Nunes Y.E., Conte-Junior C.A. (2019). Nondestructive Prediction of the Overall Quality of Cow Milk Yogurt by Correlating a Biogenic Amine Index with Traditional Quality Parameters Using Validated Nonlinear Models. J. Food Compos. Anal..

[22] Buehler A.J., Martin N.H., Boor K.J., Wiedmann M. (2018). Evaluation of Biopreservatives in Greek Yogurt to Inhibit Yeast and Mold Spoilage and Development of a Yogurt Spoilage Predictive Model. J. Dairy Sci..

[23] Nielsen L., Rolighed M., Buehler A., Knøchel S., Wiedmann M., Marvig C. (2021). Development of Predictive Models Evaluating the Spoilage-Delaying Effect of a Bioprotective Culture on Different Yeast Species in Yogurt. J. Dairy Sci..

[24] Mihaly Cozmuta A., Peter A., Mihaly Cozmuta L., Nicula C., Apjok R., Drazic G., Nzekoue F.K., Huang X., Silvi S., Sagratini G. (2020). Impact of Packaging Properties on the Physical-Chemical-Microbiological-Sensory Characteristics of Ricotta Cheese during Storage. Packag. Technol. Sci..

[25] Ricciardi E.F., Pedros-Garrido S., Papoutsis K., Lyng J.G., Conte A., Del Nobile M.A. (2020). Novel Technologies for Preserving Ricotta Cheese: Effects of Ultraviolet and near-Ultraviolet–Visible Light. Foods.

[26] Pala C., Scarano C., Venusti M., Sardo D., Casti D., Cossu F., Lamon S., Spanu V., Ibba M., Marras M. (2016). Shelf Life Evaluation of Ricotta Fresca Sheep Cheese in Modified Atmosphere Packaging. Ital. J. Food Saf..

[27] Song X., Zheng Y., Zhou X., Deng Y. (2022). Quark Cheese Processed by Dense-Phase Carbon Dioxide: Shelf-Life Evaluation and Physiochemical, Rheological, Microstructural and Volatile Properties Assessment. Foods.

[28] Quintieri L., Bugatti V., Caputo L., Vertuccio L., Gorrasi G. (2021). A Food-Grade Resin with Ldh–Salicylate to Extend Mozzarella Cheese Shelf Life. Processes.

[29] Limbo S., Pellegrino L., D’Incecco P., Gobbi S., Rosi V., Fracassetti D. (2020). Storage of Pasteurized Milk in Clear PET Bottles Combined with Light Exposure on a Retail Display Case: A Possible Strategy to Define the Shelf Life and Support a Recyclable Packaging. Food Chem..

[30] Edwards K.M., Badiger A., Heldman D.R., Klein M.S. (2021). Metabolomic Markers of Storage Temperature and Time in Pasteurized Milk. Metabolites.

[31] Gonçalves F.C., de Oliveira V.M., Martins F.T., Lião L.M., Ferri P.H., Queiroz Júnior L.H.K. (2022). Predicting Chemical Shelf Life of Mozzarella Cheese Submitted to Irregular Refrigeration Practices by Nuclear Magnetic Resonance Spectroscopy and Statistical Analysis. J. Food Compos. Anal..

[32] Lim J.Y., Lee C.L., Kim G.H., Bang Y.J., Rhim J.W., Yoon K.S. (2020). Using Lactic Acid Bacteria and Packaging with Grapefruit Seed Extract for Controlling *Listeria monocytogenes* Growth in Fresh Soft Cheese. J. Dairy Sci..

[33] Sarkar D., Ratkowsky D.A., Wang B., Bowman J.P., Tamplin M.L. (2021). Modelling Viability of *Listeria monocytogenes* in Paneer. Food Microbiol..

[34] Pal A., Bhunia K. (2025). Modeling the Biphasic and Monophasic Microbial Growth in Pasteurized Cow Milk Under Isothermal Temperature Abuse. J. Food Process Eng..

[35] Aliakbarian B., Bagnasco L., Perego P., Leardi R., Casale M. (2016). UV-VIS Spectroscopy for Monitoring Yogurt Stability during Storage Time. Anal. Methods.

[36] Bellassi P., Rocchetti G., Maldarizzi G., Braceschi G.P., Morelli L., Lucini L., Cappa F. (2021). Case Study on the Microbiological Quality, Chemical and Sensorial Profiles of Different Dairy Creams and Ricotta Cheese during Shelf-Life. Foods.

[37] Alhamdan A.M. (2022). NIR Spectroscopy Assessment of Quality Index of Fermented Milk (Laban) Drink Flavored with Date Syrup during Cold Storage. Fermentation.

[38] Guneser O. (2021). Kinetic Modelling of Betalain Stability and Color Changes in Yogurt during Storage. Pol. J. Food Nutr. Sci..

[39] Gunjal M., Khalangre A., Singh J., Ercisli S., Rasane P. (2025). Novel Approach for Development and Optimization of Microgreens-Based Functional Dairy Beverage: Antioxidant Enhancement, Consumer Acceptability, and Kinetic Shelf-Life Modeling. Food Chem. X.

[40] Singh T.P., Raigar R.K., Bam J., Paul V. (2022). Predictive Modeling for Physicochemical and Microbial Quality Assessment of Vacuum-Packed Yak Milk Paneer under Various Storage Temperatures. J. Food Process. Preserv..

[41] Chaturvedi K., Basu S., Singha S., Das K. (2023). Predictive Microbial Growth Modelling for an Effective Shelf-Life Extension Strategy of Chhana (Indian Cottage Cheese). Food Control.

[42] Coşansu S. (2018). Survival Kinetics of Heat-Stressed *Escherichia coli* O157:H7 and *Listeria monocytogenes* Cells as Post-Fermentation Contaminants in Kefir during Refrigerated Storage. LWT.

[43] Rodriguez-Martinez V., Velázquez G., de Jesús Rodríguez Altaif R., Fagotti F., Welti-Chanes J., Torres J.A. (2020). Deterministic and Probabilistic Predictive Microbiology-Based Indicator of the Listeriosis and Microbial Spoilage Risk of Pasteurized Milk Stored in Residential Refrigerators. LWT.

[44] Hough G., Langohr K., Gómez G., Curia A. (2003). Survival Analysis Applied to Sensory Shelf Life of Foods. J. Food Sci..

[45] Hwang J.H., Jung A.H., Yu S.S., Park S.H. (2022). Rapid Freshness Evaluation of Cow Milk at Different Storage Temperatures Using in Situ Electrical Conductivity Measurement. Innov. Food Sci. Emerg. Technol..

[46] Papadopoulou O.S., Argyri A.A., Kounani V., Tassou C.C., Chorianopoulos N. (2021). Use of Fourier Transform Infrared Spectroscopy for Monitoring the Shelf Life and Safety of Yogurts Supplemented with a *Lactobacillus plantarum* Strain with Probiotic Potential. Front. Microbiol..

[47] Magnaghi L.R., Zanoni C., Alberti G., Quadrelli P., Biesuz R. (2022). Towards Intelligent Packaging: BCP-EVOH@ Optode for Milk Freshness Measurement. Talanta.

[48] Peng D., Xu R., Zhou Q., Yue J., Su M., Zheng S., Li J. (2023). Discrimination of Milk Freshness Based on Synchronous Two-Dimensional Visible/Near-Infrared Correlation Spectroscopy Coupled with Chemometrics. Molecules.

[49] Pérez-Calabuig A.M., Pradana-López S., Cancilla J.C., Mena M.L., Torrecilla J.S. (2025). AI-Powered Optical Quality Control of Yogurt: Detecting Melamine Adulteration and Monitoring Shelf-Life. Appl. Food Res..

[50] Cheng S., Qin Y., Mao Y., Cao Y., Zheng R., Han J., Tian S., Qin Z. (2025). “Reference Sample Comparison Method”: A New Voltammetric Electronic Tongue Method and Its Application in Assessing the Shelf Life of Fresh Milk. Food Chem..

[51] Ziyaina M., Rasco B., Coffey T., Ünlü G., Sablani S.S. (2019). Colorimetric Detection of Volatile Organic Compounds for Shelf-Life Monitoring of Milk. Food Control.

[52] Abdanan Mehdizadeh S., Noshad M. (2024). Smartphone Application Assisted Optoelectronic Nose for Monitoring Milk Freshness. J. Food Eng..

[53] Kapse S., Kedia P., Kumar A., Kausley S., Pal P., Rai B. (2023). A Non-Invasive Method for Detection of Freshness of Packaged Milk. J. Food Eng..

[54] Rashid A., Javed I., Rasco B., Sablani S., Ayaz M., Ali M.A., Abdullah M., Imran M., Gondal T.A., Afzal M.I. (2019). Measurement of Off-Flavoring Volatile Compounds and Microbial Load as a Probable Marker for Keeping Quality of Pasteurized Milk. Appl. Sci..

[55] Sakare P., Giri S.K., Mohapatra D., Kar A., Modhera B. (2024). Addressing Spoilage Monitoring in Packaged Paneer (Indian Cottage Cheese): Lac Dye-Based Intelligent Indicator as an Innovative Solution. Food Biosci..

[56] Meng G., Zhang C., Du P., Sun S., Zhang X., Wang B., Lu X. (2022). A Dual-Channel Luminescent Signal Readout Nanoprobe for Rapid Monitoring of Biogenic Amines in Milk and Yogurt. Sens. Actuators B Chem..

[57] Shmueli G. (2010). To Explain or to Predict?. Stat. Sci..

[58] Roberts D.R., Bahn V., Ciuti S., Boyce M.S., Elith J., Guillera-Arroita G., Hauenstein S., Lahoz-Monfort J.J., Schröder B., Thuiller W. (2017). Cross-validation Strategies for Data with Temporal, Spatial, Hierarchical, or Phylogenetic Structure. Ecography.

[59] Varma S., Simon R. (2006). Bias in Error Estimation When Using Cross-Validation for Model Selection. BMC Bioinform..

[60] Mcmeekin T.A., Ross T. (1996). Modeling Applications. J. Food Prot..

[61] Brahimi N., Medouni-Haroune L., Messaoudene L., Mouhoubi K., Remini H., Allam A., Brara Z., Brahimi Y., Ouchen A., Negrichi S. (2025). Evaluation and Validation Criteria for Kinetic Models in Food Science: A Critical Review. Trends Food Sci. Technol..

[62] Calvini R., Pigani L. (2022). Toward the Development of Combined Artificial Sensing Systems for Food Quality Evaluation: A Review on the Application of Data Fusion of Electronic Noses, Electronic Tongues and Electronic Eyes. Sensors.

[63] Giannakourou M.C., Taoukis P.S. (2019). Meta-Analysis of Kinetic Parameter Uncertainty on Shelf Life Prediction in the Frozen Fruits and Vegetable Chain. Food Eng. Rev..

[64] Corradini M.G. (2018). Shelf Life of Food Products: From Open Labeling to Real-Time Measurements. Annu. Rev. Food Sci. Technol..

