# Supplementary material for: Fresh Dairy Quality in the Era of Smart Food Systems: Deterioration Pathways, Analytical Technologies, and Predictive Modeling
Source: Foods. 2026 Sep 26;15(19):3439. doi: 10.3390/foods15193439 (PMC13664348; doi:10.3390/foods15193439)
Supplement: Supplementary file 1 [file foods-15-03439-s001.zip › foods-4557129-supplementary.pdf]

Fresh Dairy Quality in the Era of Smart Food Systems: Deterioration Pathways, Analytical Technologies, and Predictive Modeling

Laíres Lima [1,2,3], Ana I. Pereira [2], Manuela Pintado [3] and Márcio Carocho [1,*]

[1] CIMO, LA SusTEC, Campus de Santa Apolónia, Bragança Polytechnic University, 5300-253 Bragança, Portugal; laires.lima@ipb.pt
[2] Research Centre in Digitalization and Intelligent Robotics (CeDRI), Associate Laboratory for Sustainability and Technology in Mountains Regions (SusTEC), Campus de Santa Apolónia, Bragança Polytechnic University, 5300-253 Bragança, Portugal; apereira@ipb.pt
[3] CBQF—Centro de Biotecnologia e Química Fina—Laboratório Associado, Escola Superior de Biotecnologia, Universidade Católica Portuguesa, Rua Diogo Botelho 1327, 4169-005 Porto, Portugal; mpintado@ucp.pt
* Correspondence: mcarocho@ipb.pt

Supplementary material

Table S1. Database-specific search strategy and Boolean search strings used to identify studies on shelf-life assessment and prediction in fresh dairy products.

Database	Search field	Publication period	Final search date	Language restriction	Document-type filter	Records retrieved	Complete Boolean search strategy
Scopus	TITLE-ABS-KEY	2015–2025	8 August 2026	English only	None applied during database search	3599	TITLE-ABS-KEY(milk OR dairy OR yogurt OR yoghurt OR kefir OR ricotta OR mozzarella OR paneer OR chhana OR chhena OR quark OR "fresh cheese" OR "cottage cheese" OR "fermented milk" OR lassi) AND TITLE-ABS-KEY("shelf life" OR "shelf-life" OR spoilage OR deterioration OR freshness OR "storage stability" OR "storage time" OR "storage temperature") AND TITLE-ABS-KEY(model* OR predict* OR kinetic* OR sensor* OR

							spectroscop*<br>OR chemometric* OR metabolom* OR "machine learning"<br>OR "artificial intelligence" OR "electronic nose"<br>OR "electronic tongue" OR "volatile compounds"<br>OR "data fusion" OR monitor*<br>)<br>AND PUBYEAR > 2014<br>AND PUBYEAR < 2026
Web of Science Core Collection	Topic (TS)	2015–2025	8 August 2026	English only	None applied during database search	3299	TS=(<br>milk OR dairy OR yogurt OR yoghurt OR kefir OR ricotta OR mozzarella<br>OR paneer OR chhana OR chhena OR quark OR "fresh cheese"<br>OR "cottage cheese" OR "fermented milk" OR lassi<br>)<br>AND TS=(<br>"shelf life" OR "shelf-life" OR spoilage OR deterioration OR freshness<br>OR "storage stability" OR "storage time" OR "storage temperature"<br>)<br>AND TS=(<br>model* OR predict* OR kinetic* OR sensor* OR spectroscop*<br>OR chemometric* OR metabolom* OR "machine learning"<br>OR "artificial intelligence" OR "electronic nose"<br>OR "electronic tongue" OR "volatile compounds"<br>OR "data fusion" OR monitor*<br>)<br>AND PY=(2015-2025)

Table S2. Evidence map of selected studies on shelf-life assessment and prediction in fresh dairy products, including analytical approaches, modeling methods, endpoints, validation, and predictive performance.

Dairy matrix	Analytical approach	Modeling framework	Model/algorithm	Prediction target	Endpoint/ failure criterion	Validation design	Predictive performance	Shelf-life relevance	Reference
Two commercial yogurt samples	Acidity, viscosity, lactic acid bacteria counts and sensory evaluation during storage at 5-35 °C	Accelerated shelf-life testing; kinetic regression with temperature dependence	Exponential quality-change models and Arrhenius equation	Shelf life at 5 °C from acidity, viscosity or sensory score	Sensory score of 60 points; associated terminal acidity/viscosity values	Compared predictions with observed/labelled shelf life under constant temperatures; no dynamic or external validation	Predicted 15.5 and 18.5 d at 5 °C for samples 1 and 2; regression coefficients generally 0.953-0.989	Direct shelf-life prediction; limited to constant-temperature conditions	[3]
Commercial dairy and plant-based yogurts (six products; dairy products are relevant to this review)	NIR spectroscopy, physicochemical measurements, sensory liking and consumer biometrics	Supervised machine-learning regression	Artificial neural networks; PCA and correlation analysis used exploratorily	Physicochemical traits from NIR; overall liking from predicted physicochemical traits	Overall liking; no storage-based failure threshold	Random split: 65% training and 35% testing; 17 training algorithms compared	Model 1 R = 0.98; Model 2 R = 0.99; testing MSE reported as 0.04 for Model 1	Indirect: predicts quality and acceptability, but does not model storage time or shelf-life failure	[8]
Minas Frescal cheese made from conventionally or ohmically	Composition, bioactive assays, fatty acids, NMR water mobility, rheology, volatiles and sensory acceptance	Comparative process study; rheological curve fitting	Six-component Burgers model fitted by nonlinear regression; multivariate/statistical comparisons	Rheological compliance parameters, not shelf life	No explicit shelf-life endpoint	Experimental treatment comparisons; no predictive shelf-life validation	Burgers-model fits R^2 = 0.982-0.999	Indirect: characterizes effects of a preservation/processing technology on fresh-cheese quality	[10]

pasteurized milk									
Milk samples spanning low, medium and high total bacterial counts	Electronic nose responses referenced to plate-count total bacterial count	Supervised classification and quantitative regression	Back-propagation multilayer perceptron artificial neural network	TBC class and quantitative TBC (log CFU/mL)	Microbial-quality classes; no explicit sensory or commercial shelf-life threshold	Separate training, testing and exclusive validation subsets; validation included an additional pasteurized milk	100% class accuracy; quantitative $R^2 > 0.99$; mean relative difference 1.1 ± 1.7% (test) and 0.4 ± 0.8% (validation)	Indirect real-time monitoring of a principal microbial spoilage indicator	[9]
Raw cow milk sampled at farm and dairy levels	Lipase/protease activity, free fatty acids, peptides, plasmin products and ionic calcium	Observational association and analysis of regional/seasonal effects	Regression/ANOVA-type statistical models and correlation analysis	Variation in enzyme activity and candidate indicators; no shelf-life prediction	No explicit acceptability or shelf-life endpoint	Multiregional and seasonal sampling; no independent predictive validation	Adjusted $R^2 = 0.29$ (farm) and 0.23 (dairy) for lipase models	Indirect: identifies raw-milk enzyme activity and ionic calcium as potential determinants of later product stability	[16]
Reconstituted whole milk inoculated with Pseudomonas putida or Pseudomonas fluorescens	Lipolytic index/free fatty acids during 96 h at 2, 4 and 8 °C	Multiple regression of storage factors	Multiple linear regression	Free-fatty-acid/lipolysis index	Sensory rancidity threshold cited as 0.25 mEq/kg; no direct shelf-life time estimated	Two independent experiments per strain; no held-out or external validation	$R^2 = 0.77$ for P. putida and 0.43 for P. fluorescens models	Indirect: models a chemical spoilage response linked to rancid flavour	[20]
Greek yogurt with	Yeast/mold counts and visible surface	Stochastic exposure	Monte Carlo simulation with	Consumers exposed to	Visible mold; fungal	Proof-of-concept	Predicted 550 ± 25.2	Direct spoilage-risk decision	[22]

or without commercial protective cultures	growth during storage at 7 °C; facility air-plate contamination data	simulation and intervention scenarios	sensitivity and what-if analyses	yogurt with visible mold growth per million cups	spoilage level ≥10^5 CFU/g	parameterization with one facility; no external model validation reported	exposed consumers per 1 million cups under baseline assumptions	model and evaluation of biopreservative strategies	
Industrial sheep-milk ricotta fresca from three dairies, packed under modified atmosphere	Microbial counts, headspace gases, pH, water activity and composition over 0-21 d	Longitudinal comparative shelf-life assessment	Descriptive statistics and comparisons among dairies/times; no predictive model	Not applicable	Microbial deterioration and presence/growth of contaminants; no single formal failure threshold stated	Three batches from each of three industrial plants; no predictive validation	Not applicable	Direct shelf-life evaluation; MAP alone did not support a 21-d shelf life under the observed hygiene conditions	[26]
Quark cheese, untreated or DPCD-treated	Microbiology, pH, proteolysis, colour, rheology, microstructure and volatile profiling	Process optimization and multivariate discrimination	Orthogonal experimental design; PCA and OPLS-DA for volatile data	Optimal DPCD conditions and discrimination of storage/treatment groups, not remaining shelf life	Control considered unsuitable at day 14 because of excessive yeast	OPLS-DA internal cross-validation; no independent shelf-life validation	Optimization model R^2 = 0.978; OPLS-DA R^2Y = 0.978 and Q^2 = 0.933	Direct preservation study; DPCD kept treated cheese within shelf life through day 14	[27]
Pasteurized milk stored on a residential	Recorded dynamic product temperatures under load, door-opening, ambient-	Deterministic and probabilistic dynamic predictive microbiology	Temperature-integrated square-root growth models with Monte Carlo	48-h cumulative growth of Listeria monocytoge	Safety/spoilage risk indicator based on predicted log	Two 48-h experimental replicates across refrigerator	Example high-load/32.2 °C scenario: L.	Indirect dynamic risk prediction under realistic domestic time-	[40]

refrigerator door shelf	temperature and use scenarios		propagation of parameter and temperature variability	nes and Pseudomonas putida	growth; no sensory endpoint	scenarios; uncertainty expressed as 5th-95th percentile intervals	monocytogenes 1.0-2.3 log and P. putida 3.4-6.6 log (5th-95th percentile)	temperature conditions	
Fresh soft cheese inoculated with Listeria monocytogenes	Pathogen counts, water activity, pH, moisture and acidity under packaging/culture treatments at 10 and 15 °C	Primary microbial growth modeling and treatment comparison	Growth-curve parameters including lag time and maximum population density	Listeria growth response under hurdle treatments	Food-safety outcome; no explicit commercial shelf-life threshold	Replicated treatment experiments at normal and abuse temperatures; no external validation	PBAT-GSE packaging extended Listeria lag time 2.7-fold versus control at 15 °C	Safety-oriented preservation study; supports hurdle strategies rather than direct shelf-life prediction	[32]
Yogurt coloured with beetroot extract	Betalain content, pH and CIE L*a*b* colour at 4, 10 and 20 °C	Chemical degradation kinetics and multivariable calibration	First-order betalain degradation; zero-order colour change; Arrhenius analysis; multiple linear regression	Betalain content and colour stability	No sensory acceptability or explicit product-failure threshold; betalain half-life reported	Goodness-of-fit on storage data; no independent validation set	Betalain kinetic R^2 = 0.969-0.983; regression R^2 = 97.55% at 4 °C and 98.59% at 10 °C; half-life 51.43 d at 4 °C	Indirect: quantifies colourant stability, not total product shelf life	[41]
Radish-microgreen lassi (fermented dairy beverage)	Bioactives, antioxidant assays, sensory acceptability, acidity, FFA, TBA and microbial	Mixture optimization plus accelerated kinetic shelf-life modeling	Response surface methodology; zero-, first- and second-order kinetics;	Quality-attribute half-lives and shelf-life estimates at 5 °C	Attribute-specific t1/2 values; microbial load used for a	Observed-versus-predicted optimization responses and fit	Zero-order models R^2 = 0.901-0.998; predicted t1/2 at 5 °C	Direct, but yields different shelf-life values by indicator rather than one	[42]

	counts at 5, 15 and 25 °C		Arrhenius, Eyring and Ball models		9.33-d estimate	statistics; no independent product-validation set	ranged 6.43-59.73 d; microbial estimate 9.33 d	integrated failure endpoint	
Vacuum-packed yak-milk paneer in two packaging films	Physicochemical properties and standard plate, yeast/mold and coliform counts at 8, 16 and 24 °C	Accelerated quality kinetics and predictive microbiology	Arrhenius/exponential quality-change models and modified Gompertz growth models	Physicochemical deterioration and microbial acceptability /shelf life	Microbial load-based acceptability; exact numerical terminal limit not clearly specified in the extracted report text	Goodness-of-fit against the experimental storage data; no external validation	Modified Gompertz $R^2 \geq 0.79$; reported microbial fits mostly 0.89-0.99 with low RMSE	Direct shelf-life prediction across packaging and temperature conditions	[43]
Fresh paneer inoculated with a five-strain Listeria monocytogenes cocktail	Pathogen counts across 4-45 °C and pH 5.0-6.0	Primary/secondary predictive microbiology and growth/no-growth modeling	Extended Ratkowsky square-root model; logistic growth/no-growth model; comparison with ComBase, PMP and FSSP	Growth rate and probability of growth of Listeria monocytogenes	Safety endpoint: growth/no growth of Listeria; not a sensory/commercial shelf-life endpoint	Independent paneer growth/no-growth experiment plus comparison with independent cheese data and published models	Secondary model R^2 = 0.9486, RMSE = 0.0348; paneer validation 93.34% correct; ComBase Bf = 1.01, Af = 1.12	Direct safety-oriented shelf-life/risk support for paneer	[33]
High-moisture mozzarella in active or	Packaging release, microbial counts, pH, proteolysis, texture and colour at 4 and 15 °C	Microbial shelf-life curve fitting	Modified Gompertz equation	Time to microbial acceptability limit	4 log CFU/g for coliforms and 6 log CFU/g for	Triplicate experimental storage curves; no independent	Model estimated approximately 2 d of shelf-life	Direct shelf-life prediction linked to active packaging	[28]

control PET trays					pseudomonads	external validation	extension at 4 °C		
Commercially prepared chhana, with or without whey immersion	Natural microbial flora and compositional changes under isothermal and dynamic storage	Primary and secondary predictive microbiology, including dynamic-temperature prediction	Logistic, Gompertz, Baranyi, Ratkowsky square-root and three-phase linearized growth model	Microbial growth and product shelf life under static/dynamic storage	Microbial-group limits used for shelf-life estimation; exact thresholds vary by group	Model validation under dynamic refrigerated storage; comparison of predicted and observed microbial counts	Primary-model R^2 mostly 0.94-0.99; secondary RMSE 0.032-0.084; prediction rates up to 96.2-96.5%	Direct shelf-life prediction and evaluation of whey immersion as an extension strategy	[38]
Pasteurized double-toned, toned and full-cream cow milk	Total plate count plus pH, proteolysis, lipolysis and titratable acidity at 4-45 °C	Primary/secondary predictive microbiology	Biphasic Baranyi model at 4 °C; Baranyi-Roberts model at 10-45 °C; Huang square-root secondary model	Microbial growth and time to expiry across temperatures	TPC > 4.47 log CFU/mL	Fit to isothermal experimental curves; no independent external validation	R^2 = 0.98-0.99 and RMSE = 0.05-0.46; shelf life at 4 °C: 168, 350 and 120 h for the three milk types	Direct shelf-life estimation, including extreme temperature-abuse scenarios	[34]
Kefir inoculated post-fermentation with heat-stressed pathogens	Pathogen survival counts during 14 d at 4 °C	Microbial inactivation/survival kinetics	Log-linear, log-linear-shoulder and Weibull models	Survival curve, shoulder and 4D reduction time	Safety endpoint: 4-log reduction time; no commercial shelf-life endpoint	Replicated curve fitting with model comparison by R^2 and RMSE; no external validation	Nonlinear models generally R^2 = 0.96-1.00 and RMSE = 0.08-0.57; 4D times varied by heat	Safety-oriented storage kinetics rather than sensory shelf-life prediction	[39]

							treatment and organism		
Commercial full-fat, fat-free and blueberry yogurts	Diffuse-reflectance UV-VIS, CIE colour, rheology and pH over 49 d at 4 °C	Chemometric storage-age prediction and multiway exploratory analysis	GA-PLS/PLS regression; Tucker3 three-way PCA	Yogurt storage age (days)	No explicit acceptability threshold; colour change became visible after the expiry date	Five-group Venetian-blinds cross-validation plus independent external test set of 16 spectra	External-test SEP = 3.3 d; bias = -0.1 d	Indirect: predicts storage age and stability rather than remaining shelf life from a defined failure endpoint	[35]
Fresh pasteurized milk stored at 4, 25, 35 and 45 °C	Voltammetric electronic tongue fingerprints with microbiological and sensory reference measurements	Reference-sample differential-degree modeling plus kinetic/Arrhenius shelf-life prediction	One-to-one differential-degree model; zero-/first-order kinetics; Arrhenius; SVM and DFA comparators	Microbiological-quality and sensory shelf life	APC limit 50,000 CFU/mL and sensory rejection reference	Thirty-six real samples at 3, 5 and 7 d; comparison against held-out SVM/DFA results	Predicted 3.4, 5.6 and 8.0 d for actual 3, 5 and 7 d (86.29-88.60% accuracy); Arrhenius fit R^2 = 0.939	Direct electronic-tongue shelf-life assessment with explicit microbiological-quality and sensory outputs	[50]
Pasteurized 2% fat milk from multiple production batches	Untargeted and targeted NMR metabolomics at 4, 10, 15 and 20 °C	Time-temperature trend and threshold analysis of candidate metabolites	Linear/nonlinear regression of metabolite trajectories; multivariate metabolomics analyses	Storage-related metabolite change and candidate time-temperature indicators	Metabolite-specific onset/threshold time; no validated consumer-rejection threshold	Multiple pasteurization batches and temperatures; no independent predictive validation	No single predictive-performance metric reported; citric acid changed linearly and several acids	Indirect: identifies molecular markers for future real-time shelf-life indicators	[30]

							showed temperature-dependent threshold times		
Vacuum-packed mozzarella under adequate or simulated irregular refrigeration	1H NMR metabolite profiling, physicochemical analysis and quantified succinate over 11 weeks	Chemometric longitudinal treatment-effect analysis	Principal response curves with Monte Carlo permutation tests	Chemical degradation state and equivalent loss of storage life	Chemical equivalence based on succinate trajectory; no sensory or microbiological failure threshold	Treatment comparison over time with 199-permutation tests; no independent validation set	Succinate at week 4 under irregular cooling matched week 8 under adequate cooling; inferred 4-week chemical shelf-life loss	Direct chemical shelf-life inference under refrigeration abuse, but endpoint is not acceptability-based	[31]
Pasteurized whole milk stored at 7, 13, 15 and 19 °C	SiO_2 nanoparticle/Schiff-reagent colorimetric sensor, SPME-GC VOCs and aerobic plate counts	Sensor-response correlation with microbial spoilage	Regression/correlation of colour difference (ΔE) with aerobic counts	Spoilage detection and remaining-shelf-life indicator response	Aerobic counts of 5-7 log CFU/mL; spoilage times 32, 60 and 84 h at 19, 15 and 13 °C	Testing across four temperatures; no independent external validation; sensor did not respond at 7 °C	ΔE versus aerobic-count R^2 = 0.81-0.96	Direct prototype for real-time package-based spoilage monitoring	[51]
Milk samples followed	Six-dye colorimetric sensor array imaged and	Supervised freshness	Decision tree classifier; curvature test for	Freshness class / total	TBC reaching 7 log CFU/g	Three-fold cross-validation	Overall evaluation error 3%;	Direct real-time freshness classification,	[52]

through microbial deterioratio n	interpreted by smartphone	classification with feature selection	sensor/feature selection	bacterial count range		plus evaluation on 20 milk samples and smartphone light calibration	zero error for most classes, but 35% error at 4-4.5 log CFU/g	without prediction of remaining time	
Cow milk stored at 5 and 20 °C	Continuous in situ electrical conductivity with pH and microbial reference counts	Empirical freshness-index regression	Second-order polynomial models linking electrical freshness index to pH and microbial groups	pH and total aerobic, psychrotrop hic and lactic acid bacterial counts	Freshness-index initiation points: day 15 at 5 °C and day 3 at 20 °C	Longitudina l fit at two temperature s; no independent validation dataset	pH R^2 = 0.938 (5 °C) and 0.860 (20 °C); microbial R^2 = 0.736-0.959, while LAB was not modeled at 5 °C	Direct rapid freshness monitoring; detects spoilage onset rather than remaining shelf-life time	[45]
Milk in plastic and glass jars	Capacitive NFC tag read by smartphone, calibrated against pH and dielectric/capacitan ce change	Physics-based threshold classification and circuit simulation	Parallel-plate capacitance model and NFC resonance design	Binary fresh/spoiled status	pH 6.3 used as the critical spoilage threshold	Experimenta l capacitance/ pH measuremen ts in two package materials plus tag simulation; no independent sample set	Plastic: 40.5-41.6 pF fresh vs 42.1-45.6 pF spoiled; glass: 36.8-40.6 vs 40.9-42.7 pF	Direct package-level freshness detection; remaining shelf life was proposed only as future work	[53]
Whole, semi-skimmed	BCP-EVOH colour optode with RGB	Chemometric classification and	PCA/three-way PCA; LDA	Fresh/spoile d class and Soxhlet-	Freshness/spo ilage classes defined from	10:7 training/test split by milk	Classificati on 97.7% correct in	Direct intelligent-packaging platform for real-	[47]

and skimmed milk during chilled spoilage	imaging; titratable acidity reference	quantitative calibration	classification; PLS regression	Henkel titratable acidity	titratable acidity	type, cross-validation, cross-matrix prediction and a fully independent unknown-sample set	cross-validation and 91.7% in prediction; general PLS RMSECV = 0.2129 and independent-set RMSEP = 0.6526 °SH	time freshness monitoring	
Control and probiotic bovine-milk yogurts, some inoculated with Listeria monocytogenes	FTIR spectra with microbiology, pH/acidity, molecular typing and sensory scores at 4 and 12 °C	Chemometric regression and freshness classification	PLS-R, SVM-R and radial-basis SVM classification	Microbial counts and sensory freshness class/score	Sensory classes: fresh, marginal and unacceptable; pathogen counts treated as safety outcomes	Independent production batch for external validation (N = 148); leave-one-out and 10-fold CV used during training	Sensory PLS-R R^2 = 0.86-0.89, RMSE = 0.25-0.29; SVM class accuracy 87.51-92.57%; Listeria prediction poor	Direct spectral monitoring of sensory shelf life plus microbial quality; safety prediction was unreliable	[46]
Milk classified as fresh, sub-fresh or spoiled during storage	Vis/NIR and synchronous 2D-Vis/NIR correlation spectra with physicochemical reference indicators	Supervised multiclass freshness discrimination	Threshold method, LDA and SVM; grid search and particle-swarm optimization	Fresh, sub-fresh or spoiled class	Three freshness states defined from storage-related reference quality measurements	Cross-validation plus a separate prediction set of 33 unknown milk samples	2D-Vis/NIR SVM accuracy >97%; RBF-SVM achieved 100% for all three	Direct freshness classification, without prediction of remaining shelf-life time	[48]

							classes in the reported model; sub-fresh external accuracy 93.3%		
Whole fresh chilled pasteurized milk stored at 4.5 °C for up to 26 d	PTR-MS VOC profiling, trained descriptive sensory analysis and total microbial numbers	Multivariate association/exploratory analysis	General linear models and multiple factor analysis	Relationships among microbes, VOCs and sensory change; no explicit predictive target	Sensory deterioration relative to fresh reference; no single numerical failure threshold	Three milk batches and trained panel; no predictive validation	Weak overall relationship among TMN, VOCs and sensory attributes; no predictive metric reported	Mechanistic/indicator evidence showing microbial counts alone do not reliably define sensory shelf life	[18]
Dairy creams and ricotta made with a 30% cream blend; ricotta followed for 12 d	UHPLC-QTOF-MS untargeted metabolomics, microbiology and sensory analysis	Supervised/unsupervised metabolomic discrimination over shelf life	PCA and OPLS-DA with VIP markers	Discrimination of cream process and ricotta storage stage; marker discovery	No explicit acceptability/failure threshold	Cross-validation ANOVA, 100-permutation tests and outlier checks; no external sample set	Cream model $R^2Y = 0.933$, $Q^2 = 0.983$; ricotta storage model goodness-of-fit 0.993 and prediction 0.801	Indirect: identifies chemical/sensory markers of ricotta ageing, not a shelf-life endpoint	[36]
Pasteurized fluid milk	HS-SPME/GC-FID VOCs,	Longitudinal indicator	Univariate statistical	Not applicable;	Sensory unacceptabilit	Measurements across	No predictive-	Direct keeping-quality evidence;	[54]

stored at 4, 7 and 10 °C for 19 d	standard/psychrotrophic counts and hedonic sensory scores	assessment and correlation	comparisons/correlations; no predictive algorithm	evaluates VOCs as spoilage/keeping-quality markers	y reported after day 16 at 7 °C and day 12 at 10 °C	three temperatures; no predictive validation	performance metric reported	supports VOCs as indicators but does not estimate remaining shelf life	
Natural, sweetened and lemon-flavoured yogurts from one brand	Standardized digital photographs under two shutter speeds	Deep-learning image classification	ResNet34 convolutional neural networks	Melamine adulteration class and freshness/past-best-before class	Freshness class based on whether yogurt was past its best-before date	80% training, 10% internal validation and 10% blind validation using images from different batches	Blind freshness accuracy 96.95% and 96.86%; >85% of classes had precision, recall and F1 >0.90	Direct classification around the labelled shelf-life boundary; controlled single-brand proof of concept	[49]
Whole pasteurized milk in clear PET bottles	Dissolved oxygen, flavins, proteolysis, GC volatiles and sensory analysis during dark storage plus controlled light exposure	Multivariate kinetic modeling of light-induced quality change	PCA-derived multivariate quality score with linear kinetic models	Quality change under cumulative light exposure	Manufacturer microbiological limit <10^4 CFU/mL; proteose-peptone criterion 900 mg/L; sensory off-flavour development	Repeated light-dose scenarios at several milk ages; no independent external validation	Linear multivariate kinetic fits r = 0.97-0.99; average rate about -0.42 PC1 score/h	Direct shelf-life support under realistic retail-light exposure, though no single integrated expiry prediction	[29]
Ricotta cheese in four commercial plastic packages	Package barrier/antimicrobial properties, pH, VOCs, microbial counts and sensory acceptability at 4 °C	Comparative shelf-life determination	Statistical comparison and correlation; no predictive model	Not applicable	Simultaneous microbiological compliance and overall-acceptability score ≥2.8	Four packaging systems followed at 0, 3, 6, 9 and 14 d; no predictive validation	Shelf life was 3 d in IT, RO and SP packages and 1 d in SLO packaging	Direct endpoint-based shelf-life determination across packaging systems	[24]

Pasteurized buffalo milk with 0.2-0.6% plant-leaf extracts	HPLC phenolics, antioxidant/antimicrobial assays, microbial counts, enzymes, acidity and sensory properties at 5 °C	Preservation treatment comparison over storage	ANOVA/statistical comparisons; no predictive model	Not applicable	Microbial, protease/lipase and acidity limits with maintained sensory properties	Dose-response treatments and controls during 16 d; no external validation	0.6% extracts extended reported shelf life from 5 to 16 d	Direct preservation-based shelf-life extension evidence	[17]
Ricotta inoculated with Pseudomonas fluorescens and packed under MAP	Microbial counts and sensory quality during 9 d at 4 °C after UV-C or NUV-vis treatment	Endpoint-based microbial and sensory shelf-life modeling	Modified Gompertz models for microbial and overall sensory quality curves	Microbiological and sensory acceptability limits	Pseudomonas threshold 7 log CFU/g; sensory overall-quality score 4	Treated and untreated inoculated samples; one light dose per technology; no external validation	Control unacceptable before 5 d; treated samples acceptable beyond 6 d; shelf life extended about 50%	Direct prediction of shelf-life extension from nonthermal surface decontamination	[25]
Milk and yogurt spiked with biogenic amines	Carbon-dot/methyl-red dual-channel fluorescence sensing	Analytical calibration	Linear fluorescence-ratio calibration	Tyramine and other biogenic-amine concentrations	No product-specific freshness or shelf-life threshold	The method was tested by adding known amounts of biogenic amines to real milk and yogurt samples and measuring their recovery. A time-	Tyramine LOD 0.01 µM; milk/yogurt recovery 82.5-105.6%; calibration R^2 about 0.993	Indirect: quantifies biogenic amines and demonstrates a time-dependent spoilage response in milk, but does not establish a product-specific acceptability threshold or predict time to failure.	[56]

						dependent milk-spoilage experiment was also conducted, but no independent validation dataset was used.			
Whole pasteurized milk stored at 7, 10, 13, 15 and 19 °C	Headspace GC VOCs, bacterial counts and protease/lipase activity	Multivariate correlation of chemical and microbial spoilage indicators	Partial least squares analysis/correlation	Association of VOCs with microbial and enzyme activity; no final prediction model	End of shelf life at 5-7 log CFU/mL; enzyme-activity ranges associated with VOC formation	Multiple storage temperatures; no independent validation	No out-of-sample predictive metric reported	Indirect: provides VOC targets for future sensors and predictive quality-loss models	[19]
Date-syrup-fortified laban fermented milk drink	NIR spectra, physicochemical traits and sensory assessment over 7 d	Composite quality-index modeling and spectral regression	Quality-index equation, PLSR and artificial neural network	Quality index and individual product properties	Seven-day regulated shelf-life period; no independently defined rejection threshold	Calibration and cross-validation; no external dataset	Quality-index/time model $R^2 = 0.977$; ANN quality-index $R^2 = 0.921$ and outperformed PLSR	Direct monitoring over the stated shelf-life period; predicts a quality index rather than time-to-failure	[37]
Packaged paneer stored at 25	Agarose/lac-dye colour indicator, headspace	Real-time class indication plus	PCA and PLS regression from	Paneer quality parameters	Microbial counts above acceptable	Storage trials at ambient and	PLS linear-model R^2 >0.80 for	Direct intelligent-packaging freshness	[55]

± 1 and 8 ± 1 °C	VOC/CO2 and physicochemical/microbial reference measures	multivariate quality prediction	L*a*b*/RGB features	and fresh/moderately fresh/spoiled class	limits; day 1 at 25 °C and day 6 at 8 °C	refrigerated temperatures; no independent validation set	all reported quality parameters except FFA; indicator ΔE correlated 0.81-0.90 with quality measures	monitoring with observable failure transition	
Cow- and goat-milk yogurts, with application to commercial products	Biogenic amines, optical and conventional physicochemical/microbial quality measures, plus published sensory scores	Nonlinear/chemometric prediction of an overall quality index	Polynomial regression and PLS-VIP models	Biogenic-amine index and overall yogurt quality	Decline in overall sensory acceptability; no single universal numerical failure threshold	Bootstrap internal validation, V-fold cross-validation and application to 16 commercial yogurt samples	Cow-yogurt optical model Rv = 0.944; sensory-to-BAI Rv = 0.869; commercial application R = 0.847, accuracy 84.06%	Indirect/direct bridge: nondestructive overall-quality monitoring during cold storage; weak for goat yogurt	[21]
Yogurt with or without a Lacticaseibacillus rhamnosus bioprotective culture	Growth of four spoilage yeasts at 7, 12 and 16 °C over 30 d	Stochastic predictive microbiology	Buchanan primary growth model feeding 100,000-run Monte Carlo simulations	Yeast spoilage probability/levels through shelf life	Yeast count of 5 log CFU/mL	Comparison with 16 months of industrial dairy spoilage	Model predicted 2-fold reduction with bioprotection vs 10-	Direct stochastic spoilage prediction with real industrial validation, but conservative/mis	[23]

						data at day 35	fold observed; 49.8% vs 98.4% predicted spoiled under model assumptions	matched effect size	
HTST pasteurized fluid milk subject to gram-negative post-pasteurization contamination	Growth characterization of 17 spoilage isolates in skim-milk broth plus commercial milk-quality data	Stochastic shelf-life and intervention simulation	Primary growth parameters embedded in a Monte Carlo model with sensitivity/what-if analyses	Bacterial counts and proportion of containers spoiled at days 7 and 10	Standard plate count >20,000 CFU/mL with PPC present	External commercial validation: 127 samples at day 7 and 145 at day 10	Day 7: predicted mean 4.8 vs observed 4.3 log CFU/mL; 66.5% vs 59.1% containers above threshold; distribution difference significant	Direct probabilistic shelf-life and intervention decision model with commercial validation	[14]
Pasteurized whole milk (3.9% fat) stored at 5, 7, 10, 13, 15 and 19 °C	Aerobic, psychrotrophic and Bacillus counts; pH, titratable acidity, lipase and protease activity	Temperature-specific quality-loss and sequential time-temperature prediction	Fitted microbial/chemical change equations combined across storage segments	Time to spoilage and remaining shelf life under constant or sequential	APC 5 log CFU/mL, supported by acidity/pH change	Model fit across six storage temperatures; no independent external	Shelf life: 30, 24 and 12 d at 5, 7 and 10 °C; 72, 36 and 24 h at 13, 15 and 19 °C	Direct remaining-shelf-life prediction under temperature abuse	[15]

				temperature s		validation reported			

Table S3. SANRA self-assessment of the revised narrative review.

Item	SANRA criterion and scoring anchor	Assessment of the present review	Score
1	Justification of the article's importance for the readership. 0: importance is not justified; 1: importance is mentioned but not explicitly justified; 2: importance is explicitly justified.	The Introduction explains why reliable shelf-life assessment is relevant to dairy science and food-quality management. It identifies the central gap between detecting storage-related changes and predicting when a fresh dairy product reaches a defined quality or safety endpoint.	2
2	Statement of concrete aims or formulation of questions. 0: no aims or questions are formulated; 1: aims are stated generally; 2: one or more concrete aims or questions are formulated.	The review states concrete aims: to examine deterioration pathways and their indicators and to evaluate kinetic, probabilistic, analytical, chemometric, and machine-learning approaches according to their ability to predict a defined acceptability endpoint. Validation, transferability, and performance under realistic storage conditions are also explicitly considered.	2
3	Description of the literature search. 0: the search strategy is not presented; 1: the literature search is described briefly; 2: the search is described in detail, including search terms and inclusion criteria.	The review reports the databases, search fields, publication period, final search date, language restriction, eligibility criteria, duplicate removal, screening process, full-text assessment, reasons for exclusion, and final number of included studies. Complete database-specific search strings are provided in Supplementary Table S1, and the study-selection process is shown in Figure 1.	2
4	Referencing. 0: key statements are not supported by references; 1: key statements are referenced inconsistently; 2: key statements are supported by references.	Key statements concerning dairy-product deterioration, analytical technologies, modeling approaches, endpoint definition, validation, and predictive performance are supported by references. The primary studies included in the synthesis are also identified through consistent numerical references in Supplementary Table S2.	2
5	Scientific reasoning. 0: the article's argument is not based on appropriate evidence; 1: appropriate evidence is introduced selectively; 2: appropriate evidence is generally present.	The scientific arguments are supported by evidence from different study designs and analytical approaches. The review distinguishes monitoring from prediction, quality endpoints from safety outcomes, model fit from predictive validity, and internal evaluation from independent validation. Limitations concerning transferability, uncertainty, batch variability, and realistic temperature histories are considered when interpreting the evidence.	2

6	Appropriate presentation of data. 0: data are presented inadequately; 1: data are frequently not presented in the most appropriate way; 2: relevant outcome data are generally presented appropriately.	Data central to the review's argument are presented in the main tables and in the study-level evidence map. Section 2.4 quantifies the prediction targets and validation levels across the 46 included studies. Supplementary Table S2 reports dairy matrix, analytical approach, model, prediction target, endpoint or failure criterion, validation design, predictive performance, and relationship with shelf-life.	2
	**SANRA sum score**		**12/12**
